# Microbial metagenomes and metatranscriptomes during a coastal phytoplankton bloom

**DOI:** 10.1038/s41597-019-0132-4

**Published:** 2019-07-22

**Authors:** Brent Nowinski, Christa B. Smith, Courtney M. Thomas, Kaitlin Esson, Roman Marin, Christina M. Preston, James M. Birch, Christopher A. Scholin, Marcel Huntemann, Alicia Clum, Brian Foster, Bryce Foster, Simon Roux, Krishnaveni Palaniappan, Neha Varghese, Supratim Mukherjee, T. B. K. Reddy, Chris Daum, Alex Copeland, I.-Min A. Chen, Natalia N. Ivanova, Nikos C. Kyrpides, Tijana Glavina del Rio, William B. Whitman, Ronald P. Kiene, Emiley A. Eloe-Fadrosh, Mary Ann Moran

**Affiliations:** 1 Department of Marine Sciences, University of Georgia, Athens, GA 30602 USA; 2 Department of Marine Sciences, University of South Alabama, Mobile, AL 36688 USA; 3 Dauphin Island Sea Lab, Dauphin Island, AL 36528 USA; 4 Monterey Bay Aquarium Research Institute, Moss Landing, CA 95039 USA; 5 Department of Energy, Joint Genome Institute, Walnut Creek, California 94598 USA; 6 Department of Microbiology, University of Georgia, Athens, GA 30602 USA

**Keywords:** Element cycles, Metagenomics, Sequencing, Microbial ecology

## Abstract

Metagenomic and metatranscriptomic time-series data covering a 52-day period in the fall of 2016 provide an inventory of bacterial and archaeal community genes, transcripts, and taxonomy during an intense dinoflagellate bloom in Monterey Bay, CA, USA. The dataset comprises 84 metagenomes (0.8 terabases), 82 metatranscriptomes (1.1 terabases), and 88 16S rRNA amplicon libraries from samples collected on 41 dates. The dataset also includes 88 18S rRNA amplicon libraries, characterizing the taxonomy of the eukaryotic community during the bloom. Accompanying the sequence data are chemical and biological measurements associated with each sample. These datasets will facilitate studies of the structure and function of marine bacterial communities during episodic phytoplankton blooms.

## Abstract (summary)

**Table:** 

Design Type(s)	transcription profiling design • sequence assembly objective • biodiversity assessment objective
Measurement Type(s)	transcription profiling assay • marine metagenome • microbial community
Technology Type(s)	RNA sequencing • DNA sequencing • amplicon sequencing
Factor Type(s)	assay protocol • temporal_instant
Sample Characteristic(s)	marine metagenome • Monterey Bay • ocean biome

Machine-accessible metadata file describing the reported data (ISA-Tab format)

## Background & Summary

In pelagic marine ecosystems, a major proportion of primary production is transformed by heterotrophic microbes on the scale of hours to days^1–3^. Much of this rapidly-processed primary production is made available in the form of dissolved organic carbon (DOC), released from phytoplankton by direct excretion or through trophic interactions. Bacterial uptake of DOC produces living biomass and regenerates inorganic nutrients^1^.

**Fig. 1:**
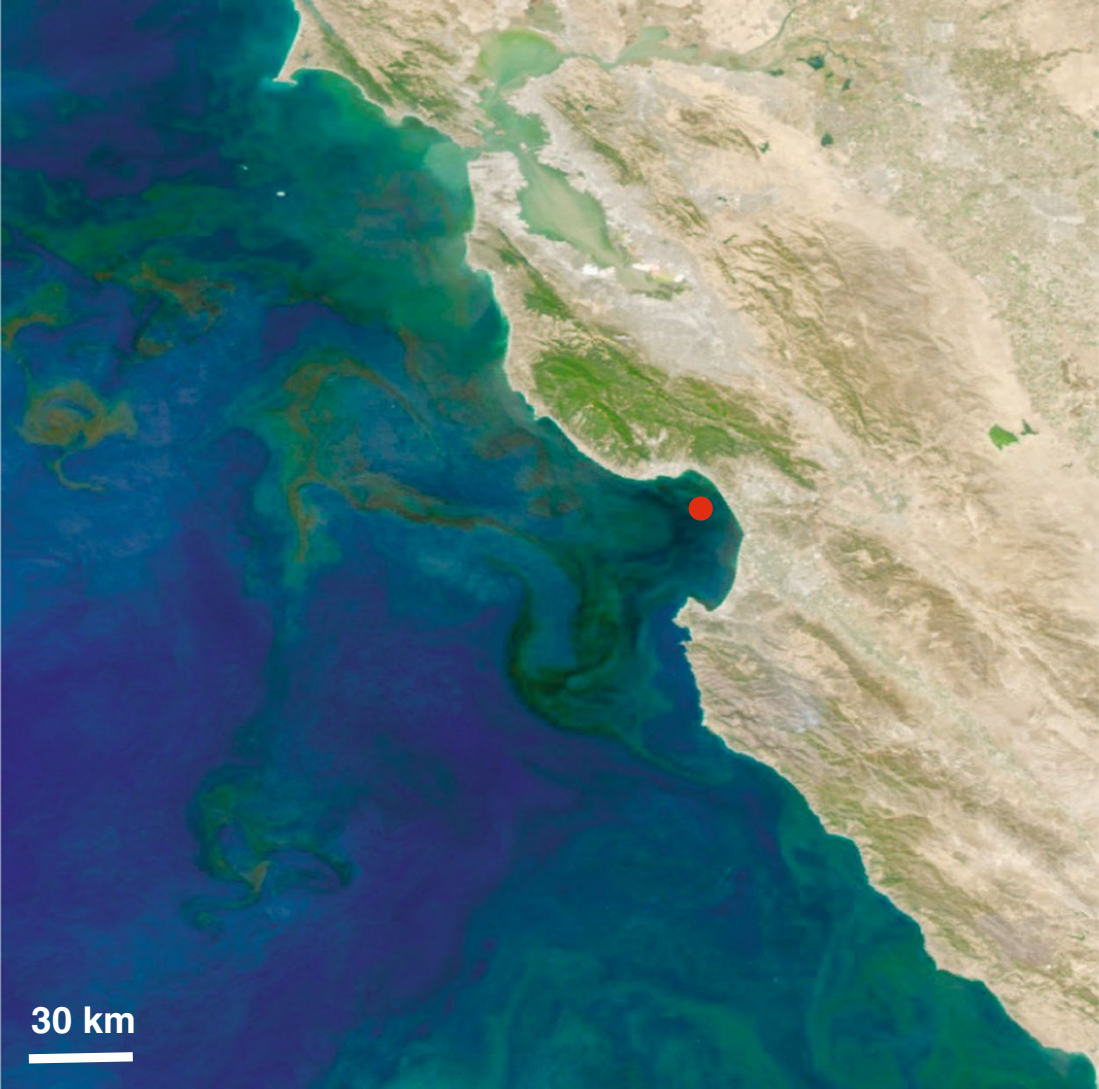
MODIS satellite image on September 26, 2016 of the phytoplankton bloom occurring in Monterey Bay and extending into the Pacific. The red dot represents the sampling station M0, located at 36.835 N, 121.901 W.

Monterey Bay is a coastal ecosystem with high primary production driven by frequent upwelling of nutrient-rich waters^4,5^. Intense phytoplankton blooms can develop^6^, and these vary dynamically in terms of taxonomic composition. In 2016, the fall phytoplankton bloom (Fig. 1) was dominated by an unusually intense bloom of the dinoflagellate *Akashiwo sanguinea*^7^. *A*. *sanguinea* cell abundances reached 4.9 × 10^6^ cells L^−1^, and chlorophyll *a* concentrations reached 57 µg L^−1^ (at ~6 m depth) over the period spanning mid-September to mid-November. Here we present metagenomic, metatranscriptomic, and iTag data on the bacterial and archaeal communities during a 52-day period spanning this unusual plankton bloom in Monterey Bay (Table 1). iTag data on the eukaryotic microbial communities provides contextual information on community dynamics of the bloom-forming phytoplankton and grazer communities.

**Table 1:** Sequence datasets from the fall bloom in Monterey Bay, CA, 2016.

Source Name	Sampling Dates	Geographical Location	Sampling Method	Sequence Type	Sample Identifiers from GOLD (Gaxxx) or the JGI Portal (Project ID xxx)	BioProject Accession IDs from the NCBI SRA
Monterey Bay Station M0	September 26 - November 16, 2016	Monterey Bay, CA, USA, 36.835 N, 121.901 W	Autonomous collection by the Environmental Sample Processor and Niskin bottle sampling	All		Umbrella project PRJNA533622
Monterey Bay Station M0	September 26 - November 16, 2016	Monterey Bay, CA, USA, 36.835 N, 121.901 W	Autonomous collection by the Environmental Sample Processor and Niskin bottle sampling	Metagenomics	Ga0228601 - Ga0228678; GA0233393 - Ga0233402	PRJNA467720 - PRJNA467773, PRJNA468208 - PRJNA468214, PRJNA502407 - PRJNA502427, PRJNA502440 - PRJNA502442
Monterey Bay Station M0	September 26 - November 16, 2016	Monterey Bay, CA, USA, 36.835 N, 121.901 W	Autonomous collection by the Environmental Sample Processor and Niskin bottle sampling	Metatranscriptomics	Ga0228679 - Ga0232167; Ga0247556 - Ga0247607; Ga0256411 - Ga0256417	PRJNA467774 - PRJNA467774, PRJNA468143 - PRJNA468143, PRJNA468299 - PRJNA468332, PRJNA502451 - PRJNA502468, PRJNA502608 - PRJNA502612
Monterey Bay Station M0	September 26 - November 16, 2016	Monterey Bay, CA, USA, 36.835 N, 121.901 W	Autonomous collection by the Environmental Sample Processor and Niskin bottle sampling	16S rRNA iTags	JGI Project ID 1190879	PRJNA511156 - PRJNA511206, PRJNA511216 - PRJNA511252
Monterey Bay Station M0	September 26 - November 16, 2016	Monterey Bay, CA, USA, 36.835 N, 121.901 W	Autonomous collection by the Environmental Sample Processor and Niskin bottle sampling	18S rRNA iTags	JGI Project ID 1190880	PRJNA511207 - PRJNA511215, PRJNA511253 - PRJNA511331

## Methods

### Sampling protocol

From September 26 through November 16, 2016, microbial cells were collected at Monterey Bay station M0 for sequence analysis. A moored autonomous robotic instrument, the Environmental Sample Processor (ESP)^8^, filtered up to 1 L of seawater sequentially through a 5.0 µm pore-size polyvinylidene fluoride filter to capture primarily eukaryotic microbes, which was stacked on top of a 0.22 µm pore-size polyvinylidene fluoride filter to capture primarily bacteria and archaea (Table 1). The samples were collected between 5 and 7 m depth at approximately 10 a.m. PST. Samples were collected daily except during October 7 – November 1 when the ESP was offline for repair. ESP filters were preserved with RNAlater at the completion of sample collection and stored in the instrument until retrieval. While the ESP was offline, grab samples were collected by Niskin bottle at the M0 mooring site 2–3 times per week, with time of sampling, depth of sampling, and filters the same as for the ESP samples except that filters were flash frozen in liquid nitrogen.

Environmental data (temperature, salinity, chlorophyll *a* fluorescence, light transmission, and dissolved O_2_ concentrations) were collected by a CTD instrument mounted with the ESP^9^. Additional environmental data were obtained from grab samples collected at the M0 mooring 2–3 times per week [total dimethylsulfoniopropionate concentration (DMSPt), dissolved DMSP concentration (DMSPd), DMSPd consumption rate, chlorophyll *a*, and cell counts by flow cytometry and microscopy]^10,11^ (Online-only Table 1).

### DNA/RNA extraction

Total community nucleic acids for metagenome, metatranscriptome, and 16S iTag sequencing were obtained from the same 0.22 µm filter (0.22–5.0 μm size fraction) using the ZymoBIOMICS DNA/RNA Miniprep Kit (Zymo Research, Irvine CA). At extraction start, internal standards were added to the lysis buffer tube (see Usage Notes), and the filter was cut into small pieces under sterile conditions to facilitate extraction. RNA was treated according to the manufacturer’s instructions with in-column DNase I treatment. After elution, RNA was treated with Turbo DNase (Invitrogen, Carlsbad CA) and concentrated using Zymo RNA Clean and Concentrator (Zymo Research). Except for a few cases of low nucleic acid yields, duplicate filters were sequenced for each sample date.

DNA for 18S rRNA gene sequencing was extracted from the 5.0 μm filters using the DNeasy Plant Mini Kit (Qiagen, Venlo NL) with modifications. Filters were cut into pieces and added into a prepared lysis tube containing ~200 µl of 1:1 mixed 0.1 and 0.5 mm zirconia/silica beads (Biospec Products, Bartlesville, OK) and 400 μl Buffer AP1. Internal standards (see Usage Notes) were added just prior to extraction. Three freeze-thaw cycles were performed using liquid nitrogen and a 65 °C water bath. Following freeze-thaw, bead beating was performed for 10 min, followed by centrifugation at 8,000 rpm for 10 min to remove foam. Following centrifugation, 45 μl of proteinase K (>600 mAU/ml, Qiagen) was added to each tube and incubated at 55 °C for 90 min with gentle rotation. Filters were then removed and the tubes incubated at 55 °C for 1 h. The DNeasy kit protocol was resumed at the RNase A addition step. Final DNA was eluted in 75 μl of diluted (1:10) TE buffer.

### Metagenome sequencing and analysis

Sequence data were generated at the Department of Energy (DOE) Joint Genome Institute (JGI) using Illumina technology. Libraries were constructed and sequenced using the HiSeq-2000 1TB platform (2 × 151 bp). For assembly, reads were trimmed and screened, and those with no mate pair were removed using BFC (v r181)^12^. Remaining reads were assembled using SPAdes (v 3.11.1)^13^. The read set was mapped to the final assembly and coverage information generated using BBMap (v 37.78)^14^ with default parameters. Assembled metagenomes were processed through the DOE JGI Metagenome Annotation Pipeline (MAP) and loaded into the *Integrated Microbial Genomes and Microbiomes* (IMG/M) platform^15,16^.

### Metatranscriptome sequencing and analysis

Sequence data were generated at the DOE JGI using Illumina technology. Libraries were constructed and sequenced using the HiSeq-2500 1TB platform (2 × 151 bp). Metatranscriptome reads were assembled using MEGAHIT (v 1.1.2)^17^. Cleaned reads were mapped to the assembly using BBMap.

### 16S and 18S iTag sequencing and analysis

Sequence data were generated at the DOE JGI using Illumina technology. Primers 515FB^18^ (5′-GTGYCAGCMGCCGCGGTAA) and 806RB^19^ (5′-GGACTACNVGGGTWTCTAAT) were used for 16S rRNA gene amplification, and primers 565F (5′-CCAGCASCYGCGGTAATTCC) and 948R (5′-ACTTTCGTTCTTGATYRA) were used for 18S rRNA gene amplification^20^. Libraries were constructed and sequenced using the Illumina MiSeq platform (2 × 301 bp). Contaminant reads were removed using the kmer filter in BBDuk, and filtered reads were processed by the JGI iTagger (v 2.2) pipeline (https://bitbucket.org/berkeleylab/jgi_itagger).

**Fig. 2:**
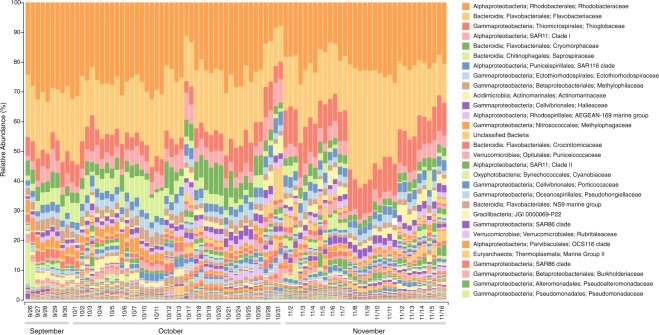
Relative abundance of bacterial and archaeal taxa at Monterey Bay station M0 during the fall of 2016. Samples were collected at ~6 m, and 16S rRNA genes were amplified from community DNA in the 0.22 to 5.0 µm size range. Taxonomic groups were defined based on exact sequence variants using DADA2 in QIIME 2 (https://qiime2.org) and assigned taxonomy with the naive Bayes q2-feature-classifier trained using the 515F/806R region from 99% operational taxonomic units from the SILVA 132 16S rRNA database. Assignments of the 30 most abundant taxa are given at the family level.

**Fig. 3:**
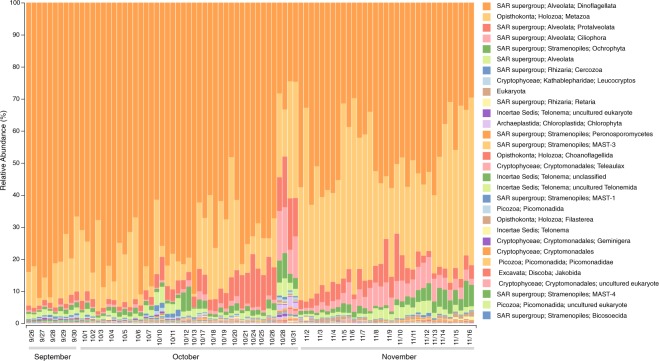
Relative abundance of eukaryotic taxa at Monterey Bay station M0 during the fall of 2016. Samples were collected at ~6 m, and 18S rRNA genes were amplified from community DNA in the >5.0 µm size range. Taxonomic groups were defined based on exact sequence variants using DADA2 in QIIME 2 (https://qiime2.org) and assigned taxonomy with the naive Bayes q2-feature-classifier trained using the 565F/948R region from 99% operational taxonomic units from the SILVA 132 18S rRNA database.

To generate an overview of microbial community composition during the bloom (Figs 2 and 3), the 16S and 18S rRNA amplicon libraries (raw reads) were primer-trimmed using Cutadapt (v 1.18)^21^ and analyzed using QIIME2 (v 2018.6)^22^. The DADA2^23^ plugin in QIIME2 was used to generate exact sequence variants (ESVs), which were classified using the QIIME2 naive Bayes classifier trained on 99% Operational Taxonomic Units (OTUs) from the SILVA rRNA database (v 132)^24^ after trimming to the primer region. Taxonomic bar plots were generated using QIIME2.

## Data Records

The raw Illumina sequencing reads for metagenomes, metatranscriptomes, and 16S rRNA and 18S rRNA iTags are available from the NCBI Sequence Read Archive under 342 separate project IDs (summarised in Online-only Table 2) which we have gathered under a single BioProject umbrella ID^25^.

Contigs assembled within each individual metagenome and metatranscriptome are available from the JGI Integrated Microbial Genomes portal (Online-only Table 2).

Chemical and biological data associated with each sample are available at the Biological and Chemical Oceanography Data Management Office (BCO-DMO)^9,10^. Measured parameters include temperature, salinity, depth, light transmission, concentrations of dissolved oxygen and chlorophyll, concentration and consumption rates of DMSP, and cell counts for heterotrophic bacteria, *Synechococcus*, *Akashiwo*, and photosynthetic eukaryotes.

## Technical Validation

For metagenomic and metatranscriptomic Illumina data, BBDuk (version 37.95; https://jgi.doe.gov/data-and-tools/bbtools/bb-tools-user-guide/bbduk-guide/) was used to remove contaminants, trim reads that contained adapter sequence, and trim reads where quality dropped to zero. BBDuk was used to remove reads that contained four or more ‘N’ bases, had an average quality score across the read <3, or had a minimum length ≤51 bp or 33% of the full read length. Reads mapped with BBMap to masked human, cat, dog and mouse references at >93% identity were separated into a chaff file. Reads aligned to common microbial contaminants were also separated into a chaff file. For metatranscriptomic data, reads containing ribosomal RNA and known JGI spike-in sequences were removed and placed into separate fastq files. The internal DNA and mRNA standards added for quantification purposes at the nucleic acid extraction step (see Usage Notes) were recovered at 0.5–5.0% of sequences as expected.

For 16S rRNA and 18S rRNA, BBDuk was used to remove contaminants and trim reads that contained adapter sequence. This program was also used to remove reads that contained one or more ‘N’ bases, had an average quality score across the read of <10, or had a minimum length ≤51 bp or 33% of the full read length. Reads mapped with BBMap to masked human, cat, dog and mouse references at >93% identity or aligned to common microbial contaminants were separated into a chaff file. The 16S and 18S rRNA reads amplified from the internal DNA standards added for quantification purposes (see Usage Notes) were recovered at their expected level (0.5–5.0% of sequences).

Sequence datasets were checked for consistency with the expected composition of coastal marine microbial communities. Taxonomic assignments of 16S and 18S rRNA ESVs matched those of marine microbes common in coastal areas in general^26,27^ and in Monterey Bay seawater in particular^11^ (Figs 2 and 3). Taxonomic assignments of protein-encoding genes from metagenomic datasets were likewise representative of coastal and Monterey Bay microbial communities, and had taxonomic assignments consistent with the iTag datasets.

## Usage Notes

Sample processing included the addition of internal standards to allow for calculation of volume-based absolute copy numbers for each gene or transcript type (i.e., counts L^−1^ rather than % of sequence library)^28,29^. The DNA standards consisted of genomic DNA from *Thermus thermophilus* DSM7039 HB8^29^ and *Blautia producta* strain VPI 4299 (American Type Culture Collection, Manassas, VA). mRNA standards consisted of custom-designed 1006 nt artificial transcripts^29^. Artificial transcript sequences are available at Addgene Plasmid Repository (https://www.addgene.org; products MTST5 and MTST6). All four standards (two DNA and two mRNA) were added to the 0.22 μm pore size samples at the initiation of nucleic acid extraction. In the case of 18S iTag samples, genomic DNA from *Arabidopsis* (BioChain Institute, Inc., Newark, CA) and *Mus musculus* (Millipore Sigma, Burlington MA) was similarly added to the 5.0 μm pore size samples at initiation of extraction. Added amounts of internal standards were estimated at ~1% of final yields of DNA or mRNA based on prior recoveries from similar filters. Actual yields averaged ~2% of reads. The internal standards should be removed from the raw data prior to analysis. Information on how internal standards can be used for volume-based quantification is available elsewhere^29,30^.

Environmental data collected in association with the nucleic acid samples are given in Online-only Table 1. Available data differ between sampling dates depending on whether sampling was done by the ESP, from Niskin grab samples, or both.

### ISA-Tab metadata file

Download metadata file

## Data Availability

Software versions and parameters used are as follows: BFC v r181 MEGAHIT v 1.1.2: –k-list 23, 43, 63, 83, 103, 123 SPAdes v 3.11.1: -m 2000, -k 33, 55, 77, 99, 127 –meta BBDuk v 38.08 for 16S, v 38.06 for 18S BBMap v 37.78 iTagger v 2.2 For 16S iTags: Cutadapt v 1.18: –interleaved -g GTGYCAGCMGCCGCGGTAA -G GGACTACNVGGGTWTCTAAT -m 275 –discard-untrimmed QIIME2 v 2018.6: qiime dada2 denoise-paired\ –p-trunc-len-f 210\ –p-trunc-len-r 181 For 18S itags: Cutadapt v 1.18: –interleaved -g CCAGCASCYGCGGTAATTCC -G ACTTTCGTTCTTGATYRA -m 275 –discard-untrimmed QIIME2 v2018.6: qiime dada2 denoise-paired\ –p-trunc-len-f 259\ –p-trunc-len-r 200

## Online-only Tables

**Online-only Table 1:** Environmental measurements associated with nucleic acid samples. An “x” indicates available data at the Biological and Chemical Data Management Office (BCO-DMO), https://www.bco-dmo.org/project/541255.

Sample Name	NCBI BioProject Accession	GOLD Project ID	Collection Date	Temperature (oC)	Salinity (psu)	Depth (m)	Dissolved Oxygen (ml/L)	Chlorophyll a (CTD) (ug/L)	Chlorophyll a (Niskin) (ug/L)	Light Transmission (%)	DMSPt (nM)	DMSPd (in situ) (nM)	DMSPd (lab) (nM)	DMSPd consumption rate (nM/d)	Photosynthetic Eukaryotes (cells /mL)	Heterotrophic Bacteria (cells/mL)	Synechococcus (cells/mL)	Akashiwo (cells/mL)
10D	PRJNA502409	Gp0266599	11/10/2016	x	x	x	x	x		x								
11D	PRJNA467725	Gp0266600	10/6/2016	x	x	x	x	x		x								
125D_r	PRJNA467773	Gp0266668	11/15/2016	x	x	x	x	x		x								
14D	PRJNA502410	Gp0266603	10/28/2016			x			x		x	x	x	x	x	x	x	x
15D	PRJNA502411	Gp0266604	10/13/2016			x			x		x		x	x	x	x	x	x
16D	PRJNA502412	Gp0266605	10/17/2016			x			x		x	x	x	x	x	x	x	x
18D	PRJNA502413	Gp0266607	10/20/2016			x			x		x	x	x		x	x	x	
1D	PRJNA467720	Gp0266591	10/4/2016	x	x	x	x	x		x								
1D_r	PRJNA502427	Gp0266664	9/26/2016	x	x	x	x	x	x	x	x	x	x	x	x	x	x	x
20D	PRJNA467726	Gp0266608	9/27/2016	x	x	x	x	x		x								
21D	PRJNA468208	Gp0272196	10/24/2016			x			x		x	x	x	x	x	x	x	x
22D	PRJNA502414	Gp0266609	10/12/2016			x			x		x	x	x	x	x	x	x	x
23D	PRJNA467727	Gp0266610	10/6/2016	x	x	x	x	x		x								
24D	PRJNA502440	Gp0272197	11/2/2016	x	x	x	x	x	x	x	x		x		x	x	x	x
25D	PRJNA502415	Gp0266611	11/6/2016	x	x	x	x	x		x								
26D	PRJNA467728	Gp0266612	10/1/2016	x	x	x	x	x		x								
27D	PRJNA468209	Gp0272198	10/12/2016			x			x		x	x	x	x	x	x	x	x
28D	PRJNA467729	Gp0266613	10/4/2016	x	x	x	x	x		x								
29D	PRJNA467730	Gp0266614	10/10/2016			x			x		x	x	x	x	x	x	x	x
2D	PRJNA467721	Gp0266592	11/16/2016	x	x	x	x	x		x								
30D	PRJNA502416	Gp0266615	10/5/2016	x	x	x	x	x	x	x	x	x	x	x	x	x	x	x
31D	PRJNA467731	Gp0266616	10/26/2016			x			x		x	x	x	x	x	x	x	x
32D	PRJNA468210	Gp0272199	10/19/2016			x			x		x	x	x	x	x	x	x	x
33D	PRJNA502417	Gp0266617	9/28/2016	x	x	x	x	x	x	x	x	x	x	x	x	x	x	x
34D	PRJNA467732	Gp0266618	9/30/2016	x	x	x	x	x	x	x	x	x	x	x	x	x	x	x
35D	PRJNA502441	Gp0272200	10/20/2016			x			x		x	x	x		x	x	x	
36D	PRJNA467733	Gp0266619	11/14/2016	x	x	x	x	x		x								
36D_r	PRJNA467770	Gp0266665	10/5/2016	x	x	x	x	x	x	x	x	x	x	x	x	x	x	x
37D	PRJNA467734	Gp0266620	10/25/2016			x			x		x	x			x	x	x	
38D	PRJNA468211	Gp0272201	10/2/2016	x	x	x	x	x		x								
39D	PRJNA467735	Gp0266621	9/28/2016	x	x	x	x	x	x	x	x	x	x	x	x	x	x	x
3D	PRJNA467722	Gp0266593	11/9/2016	x	x	x	x	x		x								
40D	PRJNA467736	Gp0266622	11/4/2016	x	x	x	x	x	x	x	x	x	x	x	x	x	x	x
41D	PRJNA467737	Gp0266623	11/13/2016	x	x	x	x	x		x								
42D	PRJNA502418	Gp0266624	9/27/2016	x	x	x	x	x		x								
43D	PRJNA468212	Gp0272202	11/8/2016	x	x	x	x	x	x	x	x	x	x	x	x	x	x	x
44D	PRJNA467738	Gp0266625	9/30/2016	x	x	x	x	x	x	x	x	x	x	x	x	x	x	x
45D	PRJNA467739	Gp0266626	11/3/2016	x	x	x	x	x		x								
46D	PRJNA468213	Gp0272203	10/21/2016			x			x		x	x	x	x	x	x	x	x
47D	PRJNA467740	Gp0266627	10/25/2016			x			x		x	x			x	x	x	
48D	PRJNA467741	Gp0266628	10/31/2016			x			x		x	x	x	x	x	x	x	
48D_r	PRJNA467771	Gp0266666	11/2/2016	x	x	x	x	x	x	x	x		x		x	x	x	x
49D	PRJNA502442	Gp0272204	10/3/2016	x	x	x	x	x	x	x	x	x	x	x	x	x	x	x
4D	PRJNA467723	Gp0266594	11/3/2016	x	x	x	x	x		x								
50D	PRJNA502419	Gp0266629	10/18/2016			x			x		x	x	x		x	x	x	
51D	PRJNA502420	Gp0266630	11/13/2016	x	x	x	x	x		x								
52D	PRJNA467742	Gp0266631	10/17/2016			x			x		x	x	x	x	x	x	x	x
53D	PRJNA502421	Gp0266632	9/29/2016	x	x	x	x	x		x								
54D	PRJNA468214	Gp0272205	11/11/2016	x	x	x	x	x		x								
55D	PRJNA502422	Gp0266633	11/8/2016	x	x	x	x	x	x	x	x	x	x	x	x	x	x	x
56D	PRJNA467743	Gp0266634	11/9/2016	x	x	x	x	x		x								
57D	PRJNA502423	Gp0266635	11/6/2016	x	x	x	x	x		x								
58D	PRJNA502424	Gp0266636	11/4/2016	x	x	x	x	x	x	x	x	x	x	x	x	x	x	x
58D_r	PRJNA467772	Gp0266667	11/4/2016	x	x	x	x	x	x	x	x	x	x	x	x	x	x	x
59D	PRJNA467744	Gp0266637	10/21/2016			x			x		x	x	x	x	x	x	x	x
60D	PRJNA502425	Gp0266638	11/12/2016	x	x	x	x	x		x								
61D	PRJNA502426	Gp0266639	10/31/2016			x			x		x	x	x	x	x	x	x	
62D	PRJNA467745	Gp0266640	10/28/2016			x			x		x	x	x	x	x	x	x	x
63D	PRJNA467746	Gp0266641	10/24/2016			x			x		x	x	x	x	x	x	x	x
64D	PRJNA467747	Gp0266642	10/5/2016	x	x	x	x	x	x	x	x	x	x	x	x	x	x	x
65D	PRJNA467748	Gp0266643	11/2/2016	x	x	x	x	x	x	x	x		x		x	x	x	x
66D	PRJNA467749	Gp0266644	10/3/2016	x	x	x	x	x	x	x	x	x	x	x	x	x	x	x
67D	PRJNA467750	Gp0266645	11/15/2016	x	x	x	x	x		x								
68D	PRJNA467751	Gp0266646	11/7/2016	x	x	x	x	x	x	x	x	x	x	x	x	x	x	
70D	PRJNA467752	Gp0266647	10/7/2016			x			x		x	x	x	x	x	x	x	x
71D	PRJNA467753	Gp0266648	10/18/2016			x			x		x	x	x		x	x	x	
73D	PRJNA467754	Gp0266649	11/16/2016	x	x	x	x	x		x								
74D	PRJNA467755	Gp0266650	11/11/2016	x	x	x	x	x		x								
75D	PRJNA467756	Gp0266651	11/14/2016	x	x	x	x	x		x								
76D	PRJNA467757	Gp0266652	11/10/2016	x	x	x	x	x		x								
77D	PRJNA467758	Gp0266653	10/7/2016			x			x		x	x	x	x	x	x	x	x
78D	PRJNA467759	Gp0266654	9/26/2016	x	x	x	x	x	x	x	x	x	x	x	x	x	x	x
79D	PRJNA467760	Gp0266655	11/7/2016	x	x	x	x	x	x	x	x	x	x	x	x	x	x	
7D	PRJNA502407	Gp0266596	10/26/2016			x			x		x	x	x	x	x	x	x	x
80D	PRJNA467761	Gp0266656	10/11/2016			x			x		x		x		x	x	x	
81D	PRJNA467762	Gp0266657	10/11/2016			x			x		x		x		x	x	x	
82D	PRJNA467763	Gp0266658	10/10/2016			x			x		x	x	x	x	x	x	x	x
84D	PRJNA467764	Gp0266659	10/11/2016			x			x		x		x		x	x	x	
85D	PRJNA467765	Gp0266660	10/13/2016			x			x		x		x	x	x	x	x	x
89D	PRJNA467766	Gp0266661	11/5/2016	x	x	x	x	x		x								
8D	PRJNA467724	Gp0266597	11/5/2016	x	x	x	x	x		x								
90D	PRJNA467768	Gp0266662	11/8/2016	x	x	x	x	x	x	x	x	x	x	x	x	x	x	x
91D	PRJNA467769	Gp0266663	11/11/2016	x	x	x	x	x		x								
9D	PRJNA502408	Gp0266598	11/15/2016	x	x	x	x	x		x								
26R	PRJNA468309	Gp0290791	10/1/2016	x	x	x	x	x		x								
58R_r	PRJNA468331	Gp0290824	11/4/2016	x	x	x	x	x	x	x	x	x	x	x	x	x	x	x
125R_r	PRJNA468332	Gp0290825	11/15/2016	x	x	x	x	x		x								
13R	PRJNA468304	Gp0290783	10/1/2016	x	x	x	x	x		x								
12R	PRJNA468303	Gp0290782	9/29/2016	x	x	x	x	x		x								
8R	PRJNA502451	Gp0290778	11/5/2016	x	x	x	x	x		x								
85R	PRJNA468329	Gp0290822	10/13/2016			x			x		x		x	x	x	x	x	x
76R	PRJNA502468	Gp0290819	11/10/2016	x	x	x	x	x		x								
40R	PRJNA468314	Gp0290799	11/4/2016	x	x	x	x	x	x	x	x	x	x	x	x	x	x	x
53R	PRJNA468320	Gp0290809	9/29/2016	x	x	x	x	x		x								
73R	PRJNA468325	Gp0290817	11/16/2016	x	x	x	x	x		x								
23R	PRJNA468307	Gp0290788	10/6/2016	x	x	x	x	x		x								
49R	PRJNA468318	Gp0290807	10/3/2016	x	x	x	x	x	x	x	x	x	x	x	x	x	x	x
17R	PRJNA468305	Gp0290786	11/12/2016	x	x	x	x	x		x								
14R	PRJNA502455	Gp0290784	10/28/2016			x			x		x	x	x	x	x	x	x	x
44R	PRJNA468315	Gp0290803	9/30/2016	x	x	x	x	x	x	x	x	x	x	x	x	x	x	x
29R	PRJNA468143	Gp0267189	10/10/2016			x			x		x	x	x	x	x	x	x	x
18R	PRJNA467774	Gp0266669	10/20/2016			x			x		x	x	x		x	x	x	
24R	PRJNA468308	Gp0290789	11/2/2016	x	x	x	x	x	x	x	x		x		x	x	x	x
79R	PRJNA468328	Gp0290821	11/7/2016	x	x	x	x	x	x	x	x	x	x	x	x	x	x	
22R	PRJNA467776	Gp0266671	10/12/2016			x			x		x	x	x	x	x	x	x	x
21R	PRJNA467775	Gp0266670	10/24/2016			x			x		x	x	x	x	x	x	x	x
60R	PRJNA502465	Gp0290813	11/12/2016	x	x	x	x	x		x								
15R	PRJNA502456	Gp0290785	10/13/2016			x			x		x		x	x	x	x	x	x
75R	PRJNA468326	Gp0290818	11/14/2016	x	x	x	x	x		x								
3R	PRJNA468301	Gp0290776	11/9/2016	x	x	x	x	x		x								
20R	PRJNA468306	Gp0290787	9/27/2016	x	x	x	x	x		x								
91R	PRJNA468330	Gp0290823	11/11/2016	x	x	x	x	x		x								
56R	PRJNA468322	Gp0290811	11/9/2016	x	x	x	x	x		x								
46R	PRJNA468316	Gp0290805	10/21/2016			x			x		x	x	x	x	x	x	x	x
2R	PRJNA468300	Gp0290775	11/16/2016	x	x	x	x	x		x								
38R	PRJNA502460	Gp0290797	10/2/2016	x	x	x	x	x		x								
45R	PRJNA502464	Gp0290804	11/3/2016	x	x	x	x	x		x								
36R	PRJNA468312	Gp0290796	11/14/2016	x	x	x	x	x		x								
39R	PRJNA468313	Gp0290798	9/28/2016	x	x	x	x	x	x	x	x	x	x	x	x	x	x	x
68R	PRJNA502467	Gp0290815	11/7/2016	x	x	x	x	x	x	x	x	x	x	x	x	x	x	
30R	PRJNA468310	Gp0290793	10/5/2016	x	x	x	x	x	x	x	x	x	x	x	x	x	x	x
48R	PRJNA468317	Gp0290806	10/31/2016			x			x		x	x	x	x	x	x	x	
42R	PRJNA502462	Gp0290801	9/27/2016	x	x	x	x	x		x								
25R	PRJNA502457	Gp0290790	11/6/2016	x	x	x	x	x		x								
78R	PRJNA468149	Gp0267195	9/26/2016	x	x	x	x	x	x	x	x	x	x	x	x	x	x	x
63R	PRJNA468147	Gp0267193	10/24/2016			x			x		x	x	x	x	x	x	x	x
43R	PRJNA502463	Gp0290802	11/8/2016	x	x	x	x	x	x	x	x	x	x	x	x	x	x	x
66R	PRJNA502466	Gp0290814	10/3/2016	x	x	x	x	x	x	x	x	x	x	x	x	x	x	x
54R	PRJNA468321	Gp0290810	11/11/2016	x	x	x	x	x		x								
41R	PRJNA502461	Gp0290800	11/13/2016	x	x	x	x	x		x								
28R	PRJNA502458	Gp0290792	10/4/2016	x	x	x	x	x		x								
32R	PRJNA468144	Gp0267190	10/19/2016			x			x		x	x	x	x	x	x	x	x
81R	PRJNA467786	Gp0266685	10/11/2016			x			x		x		x		x	x	x	
70R	PRJNA468324	Gp0290816	10/7/2016			x			x		x	x	x	x	x	x	x	x
67R	PRJNA468148	Gp0267194	11/15/2016	x	x	x	x	x		x								
33R	PRJNA502459	Gp0290794	9/28/2016	x	x	x	x	x	x	x	x	x	x	x	x	x	x	x
27R	PRJNA467777	Gp0266672	10/12/2016			x			x		x	x	x	x	x	x	x	x
34R	PRJNA468311	Gp0290795	9/30/2016	x	x	x	x	x	x	x	x	x	x	x	x	x	x	x
4R	PRJNA468302	Gp0290777	11/3/2016	x	x	x	x	x		x								
57R	PRJNA468323	Gp0290812	11/6/2016	x	x	x	x	x		x								
1R	PRJNA468299	Gp0290774	10/4/2016	x	x	x	x	x		x								
47R	PRJNA467780	Gp0266675	10/25/2016			x			x		x	x			x	x	x	
9R	PRJNA502452	Gp0290779	11/15/2016	x	x	x	x	x		x								
77R	PRJNA468327	Gp0290820	10/7/2016			x			x		x	x	x	x	x	x	x	x
55R	PRJNA468145	Gp0267191	11/8/2016	x	x	x	x	x	x	x	x	x	x	x	x	x	x	x
10R	PRJNA502453	Gp0290780	11/10/2016	x	x	x	x	x		x								
11R	PRJNA502454	Gp0290781	10/6/2016	x	x	x	x	x		x								
58R	PRJNA467783	Gp0266678	11/4/2016	x	x	x	x	x	x	x	x	x	x	x	x	x	x	x
52R	PRJNA467782	Gp0266677	10/17/2016			x			x		x	x	x	x	x	x	x	x
51R	PRJNA468319	Gp0290808	11/13/2016	x	x	x	x	x		x								
50R	PRJNA467781	Gp0266676	10/18/2016			x			x		x	x	x		x	x	x	
90R	PRJNA467788	Gp0266687	11/8/2016	x	x	x	x	x	x	x	x	x	x	x	x	x	x	x
80R	PRJNA467785	Gp0266684	10/11/2016			x			x		x		x		x	x	x	
89R	PRJNA468151	Gp0267197	11/5/2016	x	x	x	x	x		x								
35R	PRJNA467779	Gp0266674	10/20/2016			x			x		x	x	x		x	x	x	
71R	PRJNA467784	Gp0266682	10/18/2016			x			x		x	x	x		x	x	x	
31R	PRJNA467778	Gp0266673	10/26/2016			x			x		x	x	x	x	x	x	x	x
84R	PRJNA467787	Gp0266686	10/11/2016			x			x		x		x		x	x	x	
61R	PRJNA468146	Gp0267192	10/31/2016			x			x		x	x	x	x	x	x	x	
36R_r	PRJNA468152	Gp0267198	10/5/2016	x	x	x	x	x	x	x	x	x	x	x	x	x	x	x
82R	PRJNA468150	Gp0267196	10/10/2016			x			x		x	x	x	x	x	x	x	x
MB_1025D_MT	PRJNA502611	Gp0294703	10/25/2016			x			x		x	x			x	x	x	
WCR_12_MT	PRJNA502608	Gp0294698	9/26/2016	x	x	x	x	x		x								
WCR_74_MT	PRJNA502609	Gp0294699	11/6/2016	x	x	x	x	x		x								
WCR_77_MT	PRJNA502610	Gp0294700	11/7/2016	x	x	x	x	x	x	x	x	x	x	x	x	x	x	
MB_1026D_MT	PRJNA502612	Gp0294704	10/26/2016			x			x		x	x	x	x	x	x	x	x
1_DF	#N/A	#N/A	10/4/16	x	x	x	x	x		x								
3_DF	#N/A	#N/A	11/9/16	x	x	x	x	x		x								
13_DF	#N/A	#N/A	10/1/16	x	x	x	x	x		x								
26_DF	#N/A	#N/A	10/1/16	x	x	x	x	x		x								
13_S	#N/A	#N/A	10/1/16	x	x	x	x	x		x								
26_S	#N/A	#N/A	10/1/16	x	x	x	x	x		x								
38_DF	#N/A	#N/A	10/2/16	x	x	x	x	x		x								
38_S	#N/A	#N/A	10/2/16	x	x	x	x	x		x								
24_DF	#N/A	#N/A	11/2/16	x	x	x	x	x	x	x	x		x		x	x	x	x
65_DF	#N/A	#N/A	11/2/16	x	x	x	x	x	x	x	x		x		x	x	x	x
48r_DF	#N/A	#N/A	11/2/16	x	x	x	x	x	x	x	x		x		x	x	x	x
24_S	#N/A	#N/A	11/2/16	x	x	x	x	x	x	x	x		x		x	x	x	x
65_S	#N/A	#N/A	11/2/16	x	x	x	x	x	x	x	x		x		x	x	x	x
48r_S	#N/A	#N/A	11/2/16	x	x	x	x	x	x	x	x		x		x	x	x	x
49_DF	#N/A	#N/A	10/3/16	x	x	x	x	x	x	x	x	x	x	x	x	x	x	x
66_DF	#N/A	#N/A	10/3/16	x	x	x	x	x	x	x	x	x	x	x	x	x	x	x
49_S	#N/A	#N/A	10/3/16	x	x	x	x	x	x	x	x	x	x	x	x	x	x	x
66_S	#N/A	#N/A	10/3/16	x	x	x	x	x	x	x	x	x	x	x	x	x	x	x
4_DF	#N/A	#N/A	11/3/16	x	x	x	x	x		x								
45_DF	#N/A	#N/A	11/3/16	x	x	x	x	x		x								
4_S	#N/A	#N/A	11/3/16	x	x	x	x	x		x								
45_S	#N/A	#N/A	11/3/16	x	x	x	x	x		x								
28_DF	#N/A	#N/A	10/4/16	x	x	x	x	x		x								
1_S	#N/A	#N/A	10/4/16	x	x	x	x	x		x								
28_S	#N/A	#N/A	10/4/16	x	x	x	x	x		x								
40_DF	#N/A	#N/A	11/4/16	x	x	x	x	x	x	x	x	x	x	x	x	x	x	x
58_DF	#N/A	#N/A	11/4/16	x	x	x	x	x	x	x	x	x	x	x	x	x	x	x
58r_DF	#N/A	#N/A	11/4/16	x	x	x	x	x	x	x	x	x	x	x	x	x	x	x
40_S	#N/A	#N/A	11/4/16	x	x	x	x	x	x	x	x	x	x	x	x	x	x	x
58_S	#N/A	#N/A	11/4/16	x	x	x	x	x	x	x	x	x	x	x	x	x	x	x
58r_S	#N/A	#N/A	11/4/16	x	x	x	x	x	x	x	x	x	x	x	x	x	x	x
30_DF	#N/A	#N/A	10/5/16	x	x	x	x	x	x	x	x	x	x	x	x	x	x	x
64_DF	#N/A	#N/A	10/5/16	x	x	x	x	x	x	x	x	x	x	x	x	x	x	x
36r_DF	#N/A	#N/A	10/5/16	x	x	x	x	x	x	x	x	x	x	x	x	x	x	x
30_S	#N/A	#N/A	10/5/16	x	x	x	x	x	x	x	x	x	x	x	x	x	x	x
64_S	#N/A	#N/A	10/5/16	x	x	x	x	x	x	x	x	x	x	x	x	x	x	x
36r_S	#N/A	#N/A	10/5/16	x	x	x	x	x	x	x	x	x	x	x	x	x	x	x
8_DF	#N/A	#N/A	11/5/16	x	x	x	x	x		x								
89_DF	#N/A	#N/A	11/5/16	x	x	x	x	x		x								
8_S	#N/A	#N/A	11/5/16	x	x	x	x	x		x								
89_S	#N/A	#N/A	11/5/16	x	x	x	x	x		x								
11_DF	#N/A	#N/A	10/6/16	x	x	x	x	x		x								
23_DF	#N/A	#N/A	10/6/16	x	x	x	x	x		x								
11_S	#N/A	#N/A	10/6/16	x	x	x	x	x		x								
23_S	#N/A	#N/A	10/6/16	x	x	x	x	x		x								
25_DF	#N/A	#N/A	11/6/16	x	x	x	x	x		x								
57_DF	#N/A	#N/A	11/6/16	x	x	x	x	x		x								
25_S	#N/A	#N/A	11/6/16	x	x	x	x	x		x								
57_S	#N/A	#N/A	11/6/16	x	x	x	x	x		x								
70_DF	#N/A	#N/A	10/7/16			x			x		x	x	x	x	x	x	x	x
77_DF	#N/A	#N/A	10/7/16			x			x		x	x	x	x	x	x	x	x
70_S	#N/A	#N/A	10/7/16			x			x		x	x	x	x	x	x	x	x
77_S	#N/A	#N/A	10/7/16			x			x		x	x	x	x	x	x	x	x
68_DF	#N/A	#N/A	11/7/16	x	x	x	x	x	x	x	x	x	x	x	x	x	x	
79_DF	#N/A	#N/A	11/7/16	x	x	x	x	x	x	x	x	x	x	x	x	x	x	
68_S	#N/A	#N/A	11/7/16	x	x	x	x	x	x	x	x	x	x	x	x	x	x	
79_S	#N/A	#N/A	11/7/16	x	x	x	x	x	x	x	x	x	x	x	x	x	x	
43_DF	#N/A	#N/A	11/8/16	x	x	x	x	x	x	x	x	x	x	x	x	x	x	x
55_DF	#N/A	#N/A	11/8/16	x	x	x	x	x	x	x	x	x	x	x	x	x	x	x
90_DF	#N/A	#N/A	11/8/16	x	x	x	x	x	x	x	x	x	x	x	x	x	x	x
43_S	#N/A	#N/A	11/8/16	x	x	x	x	x	x	x	x	x	x	x	x	x	x	x
55_S	#N/A	#N/A	11/8/16	x	x	x	x	x	x	x	x	x	x	x	x	x	x	x
90_S	#N/A	#N/A	11/8/16	x	x	x	x	x	x	x	x	x	x	x	x	x	x	x
56_DF	#N/A	#N/A	11/9/16	x	x	x	x	x		x								
3_S	#N/A	#N/A	11/9/16	x	x	x	x	x		x								
56_S	#N/A	#N/A	11/9/16	x	x	x	x	x		x								
29_DF	#N/A	#N/A	10/10/16			x			x		x	x	x	x	x	x	x	x
82_DF	#N/A	#N/A	10/10/16			x			x		x	x	x	x	x	x	x	x
29_S	#N/A	#N/A	10/10/16			x			x		x	x	x	x	x	x	x	x
82_S	#N/A	#N/A	10/10/16			x			x		x	x	x	x	x	x	x	x
10_DF	#N/A	#N/A	11/10/16	x	x	x	x	x		x								
76_DF	#N/A	#N/A	11/10/16	x	x	x	x	x		x								
10_S	#N/A	#N/A	11/10/16	x	x	x	x	x		x								
76_S	#N/A	#N/A	11/10/16	x	x	x	x	x		x								
80_DF	#N/A	#N/A	10/11/16			x			x		x		x		x	x	x	
81_DF	#N/A	#N/A	10/11/16			x			x		x		x		x	x	x	
84_DF	#N/A	#N/A	10/11/16			x			x		x		x		x	x	x	
80_S	#N/A	#N/A	10/11/16			x			x		x		x		x	x	x	
81_S	#N/A	#N/A	10/11/16			x			x		x		x		x	x	x	
84_S	#N/A	#N/A	10/11/16			x			x		x		x		x	x	x	
54_DF	#N/A	#N/A	11/11/16	x	x	x	x	x		x								
74_DF	#N/A	#N/A	11/11/16	x	x	x	x	x		x								
91_DF	#N/A	#N/A	11/11/16	x	x	x	x	x		x								
54_S	#N/A	#N/A	11/11/16	x	x	x	x	x		x								
74_S	#N/A	#N/A	11/11/16	x	x	x	x	x		x								
91_S	#N/A	#N/A	11/11/16	x	x	x	x	x		x								
22_DF	#N/A	#N/A	10/12/16			x			x		x	x	x	x	x	x	x	x
27_DF	#N/A	#N/A	10/12/16			x			x		x	x	x	x	x	x	x	x
22_S	#N/A	#N/A	10/12/16			x			x		x	x	x	x	x	x	x	x
27_S	#N/A	#N/A	10/12/16			x			x		x	x	x	x	x	x	x	x
17_DF	#N/A	#N/A	11/12/16	x	x	x	x	x		x								
60_DF	#N/A	#N/A	11/12/16	x	x	x	x	x		x								
17_S	#N/A	#N/A	11/12/16	x	x	x	x	x		x								
60_S	#N/A	#N/A	11/12/16	x	x	x	x	x		x								
15_DF	#N/A	#N/A	10/13/16			x			x		x		x	x	x	x	x	x
85_DF	#N/A	#N/A	10/13/16			x			x		x		x	x	x	x	x	x
15_S	#N/A	#N/A	10/13/16			x			x		x		x	x	x	x	x	x
85_S	#N/A	#N/A	10/13/16			x			x		x		x	x	x	x	x	x
41_DF	#N/A	#N/A	11/13/16	x	x	x	x	x		x								
51_DF	#N/A	#N/A	11/13/16	x	x	x	x	x		x								
41_S	#N/A	#N/A	11/13/16	x	x	x	x	x		x								
51_S	#N/A	#N/A	11/13/16	x	x	x	x	x		x								
36_DF	#N/A	#N/A	11/14/16	x	x	x	x	x		x								
75_DF	#N/A	#N/A	11/14/16	x	x	x	x	x		x								
36_S	#N/A	#N/A	11/14/16	x	x	x	x	x		x								
75_S	#N/A	#N/A	11/14/16	x	x	x	x	x		x								
9_DF	#N/A	#N/A	11/15/16	x	x	x	x	x		x								
67_DF	#N/A	#N/A	11/15/16	x	x	x	x	x		x								
125r_DF	#N/A	#N/A	11/15/16	x	x	x	x	x		x								
9_S	#N/A	#N/A	11/15/16	x	x	x	x	x		x								
67_S	#N/A	#N/A	11/15/16	x	x	x	x	x		x								
125r_S	#N/A	#N/A	11/15/16	x	x	x	x	x		x								
73_DF	#N/A	#N/A	11/16/16	x	x	x	x	x		x								
2_S	#N/A	#N/A	11/16/16	x	x	x	x	x		x								
73_S	#N/A	#N/A	11/16/16	x	x	x	x	x		x								
2_DF	#N/A	#N/A	11/16/16	x	x	x	x	x		x								
16_DF	#N/A	#N/A	10/17/16			x			x		x	x	x	x	x	x	x	x
52_DF	#N/A	#N/A	10/17/16			x			x		x	x	x	x	x	x	x	x
16_S	#N/A	#N/A	10/17/16			x			x		x	x	x	x	x	x	x	x
52_S	#N/A	#N/A	10/17/16			x			x		x	x	x	x	x	x	x	x
50_DF	#N/A	#N/A	10/18/16			x			x		x	x	x		x	x	x	
71_DF	#N/A	#N/A	10/18/16			x			x		x	x	x		x	x	x	
50_S	#N/A	#N/A	10/18/16			x			x		x	x	x		x	x	x	
71_S	#N/A	#N/A	10/18/16			x			x		x	x	x		x	x	x	
5_DF	#N/A	#N/A	10/19/16			x			x		x	x	x	x	x	x	x	x
32_DF	#N/A	#N/A	10/19/16			x			x		x	x	x	x	x	x	x	x
5_S	#N/A	#N/A	10/19/16			x			x		x	x	x	x	x	x	x	x
32_S	#N/A	#N/A	10/19/16			x			x		x	x	x	x	x	x	x	x
18_DF	#N/A	#N/A	10/20/16			x			x		x	x	x		x	x	x	
35_DF	#N/A	#N/A	10/20/16			x			x		x	x	x		x	x	x	
18_S	#N/A	#N/A	10/20/16			x			x		x	x	x		x	x	x	
35_S	#N/A	#N/A	10/20/16			x			x		x	x	x		x	x	x	
46_DF	#N/A	#N/A	10/21/16			x			x		x	x	x	x	x	x	x	x
59_DF	#N/A	#N/A	10/21/16			x			x		x	x	x	x	x	x	x	x
46_S	#N/A	#N/A	10/21/16			x			x		x	x	x	x	x	x	x	x
59_S	#N/A	#N/A	10/21/16			x			x		x	x	x	x	x	x	x	x
21_DF	#N/A	#N/A	10/24/16			x			x		x	x	x	x	x	x	x	x
63_DF	#N/A	#N/A	10/24/16			x			x		x	x	x	x	x	x	x	x
21_S	#N/A	#N/A	10/24/16			x			x		x	x	x	x	x	x	x	x
63_S	#N/A	#N/A	10/24/16			x			x		x	x	x	x	x	x	x	x
37_DF	#N/A	#N/A	10/25/16			x			x		x	x			x	x	x	
47_DF	#N/A	#N/A	10/25/16			x			x		x	x			x	x	x	
37_S	#N/A	#N/A	10/25/16			x			x		x	x			x	x	x	
47_S	#N/A	#N/A	10/25/16			x			x		x	x			x	x	x	
78_DF	#N/A	#N/A	9/26/16	x	x	x	x	x	x	x	x	x	x	x	x	x	x	x
1r_DF	#N/A	#N/A	9/26/16	x	x	x	x	x	x	x	x	x	x	x	x	x	x	x
78_S	#N/A	#N/A	9/26/16	x	x	x	x	x	x	x	x	x	x	x	x	x	x	x
1r_S	#N/A	#N/A	9/26/16	x	x	x	x	x	x	x	x	x	x	x	x	x	x	x
7_DF	#N/A	#N/A	10/26/16			x			x		x	x	x	x	x	x	x	x
31_DF	#N/A	#N/A	10/26/16			x			x		x	x	x	x	x	x	x	x
7_S	#N/A	#N/A	10/26/16			x			x		x	x	x	x	x	x	x	x
31_S	#N/A	#N/A	10/26/16			x			x		x	x	x	x	x	x	x	x
20_DF	#N/A	#N/A	9/27/16	x	x	x	x	x		x								
42_DF	#N/A	#N/A	9/27/16	x	x	x	x	x		x								
20_S	#N/A	#N/A	9/27/16	x	x	x	x	x		x								
42_S	#N/A	#N/A	9/27/16	x	x	x	x	x		x								
33_DF	#N/A	#N/A	9/28/16	x	x	x	x	x	x	x	x	x	x	x	x	x	x	x
39_DF	#N/A	#N/A	9/28/16	x	x	x	x	x	x	x	x	x	x	x	x	x	x	x
33_S	#N/A	#N/A	9/28/16	x	x	x	x	x	x	x	x	x	x	x	x	x	x	x
39_S	#N/A	#N/A	9/28/16	x	x	x	x	x	x	x	x	x	x	x	x	x	x	x
14_DF	#N/A	#N/A	10/28/16			x			x		x	x	x	x	x	x	x	x
62_DF	#N/A	#N/A	10/28/16			x			x		x	x	x	x	x	x	x	x
14_S	#N/A	#N/A	10/28/16			x			x		x	x	x	x	x	x	x	x
62_S	#N/A	#N/A	10/28/16			x			x		x	x	x	x	x	x	x	x
12_DF	#N/A	#N/A	9/29/16	x	x	x	x	x		x								
53_DF	#N/A	#N/A	9/29/16	x	x	x	x	x		x								
12_S	#N/A	#N/A	9/29/16	x	x	x	x	x		x								
53_S	#N/A	#N/A	9/29/16	x	x	x	x	x		x								
34_DF	#N/A	#N/A	9/30/16	x	x	x	x	x	x	x	x	x	x	x	x	x	x	x
44_DF	#N/A	#N/A	9/30/16	x	x	x	x	x	x	x	x	x	x	x	x	x	x	x
34_S	#N/A	#N/A	9/30/16	x	x	x	x	x	x	x	x	x	x	x	x	x	x	x
44_S	#N/A	#N/A	9/30/16	x	x	x	x	x	x	x	x	x	x	x	x	x	x	x
48_DF	#N/A	#N/A	10/31/16			x			x		x	x	x	x	x	x	x	
61_DF	#N/A	#N/A	10/31/16			x			x		x	x	x	x	x	x	x	
48_S	#N/A	#N/A	10/31/16			x			x		x	x	x	x	x	x	x	
61_S	#N/A	#N/A	10/31/16			x			x		x	x	x	x	x	x	x	

**Online-only Table 2:** DNA and RNA sample information.

Sample Name	env_biome	env_feature	env_material	geo_loc_name	Collection Date	Latitude and Longitude	Volume Seawater Filtered (ml)	Type	JGI Project ID	JGI Sequencing Project ID	JGI Sequencing Project Name	JGI Sample ID	JGI Contigs Link	NCBI Project ID	NCBI BioProject Accession	NCBI SRA Accession ID	NCBI BioSample Accession	GOLD Project ID	Instrument	Sequencing Run Mode	Total Bases	Assembly Method
10D	ocean_biome	ocean	water	Monterey Bay, CA, USA	11/10/2016	36.835 N, 121.901 W	350	Metagenome	1174520	1174688	10D metaG	166744	https://genome.jgi.doe.gov/portal/10DmetaG_FD/10DmetaG_FD.download.html	502409	PRJNA502409	SRP174084	SAMN10350899	Gp0266599	Illumina HiSeq-2000	2 × 151	10,341,052,894	SPAdes assembler 3.11.1
10R	ocean_biome	ocean	water	Monterey Bay, CA, USA	11/10/2016	36.835 N, 121.901 W	350	Metatranscriptome	1174786	1174930	10R metaT	166830	https://genome.jgi.doe.gov/portal/10RmetaT_FD/10RmetaT_FD.download.html	502453	PRJNA502453	SRP174124	SAMN10350900	Gp0290780	Illumina HiSeq-2000	2 × 151	11,461,972,100	MEGAHIT v1.1.2
11D	ocean_biome	ocean	water	Monterey Bay, CA, USA	10/6/2016	36.835 N, 121.901 W	250	Metagenome	1174522	1174689	11D metaG	166745	https://genome.jgi.doe.gov/portal/11DmetaG_FD/11DmetaG_FD.download.html	467725	PRJNA467725	SRP155249	SAMN09201852	Gp0266600	Illumina HiSeq-2000	2 × 151	19,576,899,642	SPAdes assembler 3.11.1
11R	ocean_biome	ocean	water	Monterey Bay, CA, USA	10/6/2016	36.835 N, 121.901 W	250	Metatranscriptome	1174789	1174931	11R metaT	166831	https://genome.jgi.doe.gov/portal/11RmetaT_FD/11RmetaT_FD.download.html	502454	PRJNA502454	SRP174126	SAMN10351123	Gp0290781	Illumina HiSeq-2000	2 × 151	11,561,507,374	MEGAHIT v1.1.2
125D_r	ocean_biome	ocean	water	Monterey Bay, CA, USA	11/15/2016	36.835 N, 121.901 W	950	Metagenome	1174678	1174767	125D_r metaG	166823	https://genome.jgi.doe.gov/portal/125D_rmetaG_FD/125D_rmetaG_FD.download.html	467773	PRJNA467773	SRP155430	SAMN09202115	Gp0266668	Illumina HiSeq-2000	2 × 151	6,328,886,556	SPAdes assembler 3.11.1
125R_r	ocean_biome	ocean	water	Monterey Bay, CA, USA	11/15/2016	36.835 N, 121.901 W	950	Metatranscriptome	1174921	1174975	125R_r metaT	166875	https://genome.jgi.doe.gov/portal/125R_rmetaT_FD/125R_rmetaT_FD.download.html	468332	PRJNA468332	SRP163296	SAMN09200099	Gp0290825	Illumina HiSeq-2000	2 × 151	7,601,810,818	MEGAHIT v1.1.2
12R	ocean_biome	ocean	water	Monterey Bay, CA, USA	9/29/2016	36.835 N, 121.901 W	125	Metatranscriptome	1174792	1174932	12R metaT	166832	https://genome.jgi.doe.gov/portal/12RmetaT_FD/12RmetaT_FD.download.html	468303	PRJNA468303	SRP163181	SAMN09201820	Gp0290782	Illumina HiSeq-2000	2 × 151	7,804,474,562	MEGAHIT v1.1.2
13R	ocean_biome	ocean	water	Monterey Bay, CA, USA	10/1/2016	36.835 N, 121.901 W	125	Metatranscriptome	1174795	1174933	13R metaT	166833	https://genome.jgi.doe.gov/portal/13RmetaT_FD/13RmetaT_FD.download.html	468304	PRJNA468304	SRP163185	SAMN09202081	Gp0290783	Illumina HiSeq-2000	2 × 151	7,778,654,770	MEGAHIT v1.1.2
14D	ocean_biome	ocean	water	Monterey Bay, CA, USA	10/28/2016	36.835 N, 121.901 W	250	Metagenome	1174528	1174692	14D metaG	166748	https://genome.jgi.doe.gov/portal/14DmetaG_FD/14DmetaG_FD.download.html	502410	PRJNA502410	SRP174083	SAMN10350898	Gp0266603	Illumina HiSeq-2000	2 × 151	8,488,758,242	SPAdes assembler 3.11.1
14R	ocean_biome	ocean	water	Monterey Bay, CA, USA	10/28/2016	36.835 N, 121.901 W	250	Metatranscriptome	1174798	1174934	14R metaT	166834	https://genome.jgi.doe.gov/portal/14RmetaT_FD/14RmetaT_FD.download.html	502455	PRJNA502455	SRP173017	SAMN10350972	Gp0290784	Illumina HiSeq-2000	2 × 151	8,659,605,984	MEGAHIT v1.1.2
15D	ocean_biome	ocean	water	Monterey Bay, CA, USA	10/13/2016	36.835 N, 121.901 W	100	Metagenome	1174530	1174693	15D metaG	166749	https://genome.jgi.doe.gov/portal/15DmetaG_FD/15DmetaG_FD.download.html	502411	PRJNA502411	SRP174080	SAMN10351355	Gp0266604	Illumina HiSeq-2000	2 × 151	7,691,258,084	SPAdes assembler 3.11.1
15R	ocean_biome	ocean	water	Monterey Bay, CA, USA	10/13/2016	36.835 N, 121.901 W	100	Metatranscriptome	1174801	1174935	15R metaT	166835	https://genome.jgi.doe.gov/portal/15RmetaT_FD/15RmetaT_FD.download.html	502456	PRJNA502456	SRP173008	SAMN10350971	Gp0290785	Illumina HiSeq-2000	2 × 151	9,108,232,722	MEGAHIT v1.1.2
16D	ocean_biome	ocean	water	Monterey Bay, CA, USA	10/17/2016	36.835 N, 121.901 W	200	Metagenome	1174532	1174694	16D metaG	166750	https://genome.jgi.doe.gov/portal/16DmetaG_FD/16DmetaG_FD.download.html	502412	PRJNA502412	SRP174089	SAMN10350897	Gp0266605	Illumina HiSeq-2000	2 × 151	9,245,335,890	SPAdes assembler 3.11.1
17R	ocean_biome	ocean	water	Monterey Bay, CA, USA	11/12/2016	36.835 N, 121.901 W	400	Metatranscriptome	1174804	1174936	17R metaT	166836	https://genome.jgi.doe.gov/portal/17RmetaT_FD/17RmetaT_FD.download.html	468305	PRJNA468305	SRP163182	SAMN09201819	Gp0290786	Illumina HiSeq-2000	2 × 151	8,651,241,792	MEGAHIT v1.1.2
18D	ocean_biome	ocean	water	Monterey Bay, CA, USA	10/20/2016	36.835 N, 121.901 W	200	Metagenome	1174536	1174696	18D metaG	166752	https://genome.jgi.doe.gov/portal/18DmetaG_FD/18DmetaG_FD.download.html	502413	PRJNA502413	SRP174085	SAMN10351120	Gp0266607	Illumina HiSeq-2000	2 × 151	8,004,892,332	SPAdes assembler 3.11.1
18R	ocean_biome	ocean	water	Monterey Bay, CA, USA	10/20/2016	36.835 N, 121.901 W	200	Metatranscriptome	1174985	1175087	18R metaT	166879	https://genome.jgi.doe.gov/portal/18RmetaT_FD/18RmetaT_FD.download.html	467774	PRJNA467774	SRP155442	SAMN09201439	Gp0266669	Illumina HiSeq-2000	2 × 151	8,874,292,952	MEGAHIT v1.1.2
1D	ocean_biome	ocean	water	Monterey Bay, CA, USA	10/4/2016	36.835 N, 121.901 W	200	Metagenome	1174504	1174680	1D metaG	166736	https://genome.jgi.doe.gov/portal/1D_rmetaG_FD/1D_rmetaG_FD.download.html	467720	PRJNA467720	SRP155234	SAMN09201680	Gp0266591	Illumina HiSeq-2000	2 × 151	6,639,302,994	SPAdes assembler 3.11.1
1D_r	ocean_biome	ocean	water	Monterey Bay, CA, USA	9/26/2016	36.835 N, 121.901 W	95.1	Metagenome	1174670	1174763	1D_r metaG	166819	https://genome.jgi.doe.gov/portal/1DmetaG_FD/1DmetaG_FD.download.html	502427	PRJNA502427	SRP174125	SAMN10351067	Gp0266664	Illumina HiSeq-2000	2 × 151	38,554,088,892	SPAdes assembler 3.11.1
1R	ocean_biome	ocean	water	Monterey Bay, CA, USA	10/4/2016	36.835 N, 121.901 W	200	Metatranscriptome	1174768	1174924	1R metaT	166824	https://genome.jgi.doe.gov/portal/1RmetaT_FD/1RmetaT_FD.download.html	468299	PRJNA468299	SRP155435	SAMN09201822	Gp0290774	Illumina HiSeq-2000	2 × 151	10,940,653,962	MEGAHIT v1.1.2
20D	ocean_biome	ocean	water	Monterey Bay, CA, USA	9/27/2016	36.835 N, 121.901 W	73.5	Metagenome	1174538	1174697	20D metaG	166753	https://genome.jgi.doe.gov/portal/20DmetaG_FD/20DmetaG_FD.download.html	467726	PRJNA467726	SRP155254	SAMN09202066	Gp0266608	Illumina HiSeq-2000	2 × 151	13,033,898,710	SPAdes assembler 3.11.1
20R	ocean_biome	ocean	water	Monterey Bay, CA, USA	9/27/2016	36.835 N, 121.901 W	73.5	Metatranscriptome	1174807	1174937	20R metaT	166837	https://genome.jgi.doe.gov/portal/20RmetaT_FD/20RmetaT_FD.download.html	468306	PRJNA468306	SRP163187	SAMN09202082	Gp0290787	Illumina HiSeq-2000	2 × 151	9,121,070,440	MEGAHIT v1.1.2
21D	ocean_biome	ocean	water	Monterey Bay, CA, USA	10/24/2016	36.835 N, 121.901 W	250	Metagenome	1174540	1174698	21D metaG	166754	https://genome.jgi.doe.gov/portal/21DmetaG_FD/21DmetaG_FD.download.html	468208	PRJNA468208	SRP155253	SAMN09201605	Gp0272196	Illumina HiSeq-2000	2 × 151	7,000,627,270	SPAdes assembler 3.11.1
21R	ocean_biome	ocean	water	Monterey Bay, CA, USA	10/24/2016	36.835 N, 121.901 W	250	Metatranscriptome	1174988	1175088	21R metaT	166880	https://genome.jgi.doe.gov/portal/21RmetaT_FD/21RmetaT_FD.download.html	467775	PRJNA467775	SRP155445	SAMN09201531	Gp0266670	Illumina HiSeq-2000	2 × 151	9,023,160,530	MEGAHIT v1.1.2
22D	ocean_biome	ocean	water	Monterey Bay, CA, USA	10/12/2016	36.835 N, 121.901 W	100	Metagenome	1174542	1174699	22D metaG	166755	https://genome.jgi.doe.gov/portal/22DmetaG_FD/22DmetaG_FD.download.html	502414	PRJNA502414	SRP174086	SAMN10350896	Gp0266609	Illumina HiSeq-2000	2 × 151	7,011,736,038	SPAdes assembler 3.11.1
22R	ocean_biome	ocean	water	Monterey Bay, CA, USA	10/12/2016	36.835 N, 121.901 W	100	Metatranscriptome	1174991	1175089	22R metaT	166881	https://genome.jgi.doe.gov/portal/22RmetaT_FD/22RmetaT_FD.download.html	467776	PRJNA467776	SRP155447	SAMN09202116	Gp0266671	Illumina HiSeq-2000	2 × 151	8,949,209,488	MEGAHIT v1.1.2
23D	ocean_biome	ocean	water	Monterey Bay, CA, USA	10/6/2016	36.835 N, 121.901 W	150	Metagenome	1174544	1174700	23D metaG	166756	https://genome.jgi.doe.gov/portal/23DmetaG_FD/23DmetaG_FD.download.html	467727	PRJNA467727	SRP155978	SAMN09201453	Gp0266610	Illumina HiSeq-2000	2 × 151	7,254,602,022	SPAdes assembler 3.11.1
23R	ocean_biome	ocean	water	Monterey Bay, CA, USA	10/6/2016	36.835 N, 121.901 W	150	Metatranscriptome	1174810	1174938	23R metaT	166838	https://genome.jgi.doe.gov/portal/23RmetaT_FD/23RmetaT_FD.download.html	468307	PRJNA468307	SRP163179	SAMN09201818	Gp0290788	Illumina HiSeq-2000	2 × 151	8,584,843,770	MEGAHIT v1.1.2
24D	ocean_biome	ocean	water	Monterey Bay, CA, USA	11/2/2016	36.835 N, 121.901 W	425	Metagenome	1174546	1174701	24D metaG	166757	https://genome.jgi.doe.gov/portal/24DmetaG_FD/24DmetaG_FD.download.html	502440	PRJNA502440	SRP174090	SAMN10351047	Gp0272197	Illumina HiSeq-2000	2 × 151	6,136,433,734	SPAdes assembler 3.11.1
24R	ocean_biome	ocean	water	Monterey Bay, CA, USA	11/2/2016	36.835 N, 121.901 W	425	Metatranscriptome	1174813	1174939	24R metaT	166839	https://genome.jgi.doe.gov/portal/24RmetaT_FD/24RmetaT_FD.download.html	468308	PRJNA468308	SRP163186	SAMN09201817	Gp0290789	Illumina HiSeq-2000	2 × 151	8,896,620,114	MEGAHIT v1.1.2
25D	ocean_biome	ocean	water	Monterey Bay, CA, USA	11/6/2016	36.835 N, 121.901 W	575	Metagenome	1174548	1174702	25D metaG	166758	https://genome.jgi.doe.gov/portal/25DmetaG_FD/25DmetaG_FD.download.html	502415	PRJNA502415	SRP174108	SAMN10351090	Gp0266611	Illumina HiSeq-2000	2 × 151	7,480,494,700	SPAdes assembler 3.11.1
25R	ocean_biome	ocean	water	Monterey Bay, CA, USA	11/6/2016	36.835 N, 121.901 W	575	Metatranscriptome	1174816	1174940	25R metaT	166840	https://genome.jgi.doe.gov/portal/25RmetaT_FD/25RmetaT_FD.download.html	502457	PRJNA502457	SRP174129	SAMN10351332	Gp0290790	Illumina HiSeq-2000	2 × 151	10,195,628,720	MEGAHIT v1.1.2
26D	ocean_biome	ocean	water	Monterey Bay, CA, USA	10/1/2016	36.835 N, 121.901 W	125	Metagenome	1174550	1174703	26D metaG	166759	https://genome.jgi.doe.gov/portal/26DmetaG_FD/26DmetaG_FD.download.html	467728	PRJNA467728	SRP155258	SAMN09201452	Gp0266612	Illumina HiSeq-2000	2 × 151	7,294,852,280	SPAdes assembler 3.11.1
26R	ocean_biome	ocean	water	Monterey Bay, CA, USA	10/1/2016	36.835 N, 121.901 W	125	Metatranscriptome	1174819	1174941	26R metaT	166841	https://genome.jgi.doe.gov/portal/26RmetaT_FD/26RmetaT_FD.download.html	468309	PRJNA468309	SRP163184	SAMN09202122	Gp0290791	Illumina HiSeq-2000	2 × 151	6,828,158,996	MEGAHIT v1.1.2
27D	ocean_biome	ocean	water	Monterey Bay, CA, USA	10/12/2016	36.835 N, 121.901 W	100	Metagenome	1174552	1174704	27D metaG	166760	https://genome.jgi.doe.gov/portal/27DmetaG_FD/27DmetaG_FD.download.html	468209	PRJNA468209	SRP155263	SAMN09202468	Gp0272198	Illumina HiSeq-2000	2 × 151	8,451,209,978	SPAdes assembler 3.11.1
27R	ocean_biome	ocean	water	Monterey Bay, CA, USA	10/12/2016	36.835 N, 121.901 W	100	Metatranscriptome	1174994	1175090	27R metaT	166882	https://genome.jgi.doe.gov/portal/27RmetaT_FD/27RmetaT_FD.download.html	467777	PRJNA467777	SRP155449	SAMN09202117	Gp0266672	Illumina HiSeq-2000	2 × 151	10,817,948,644	MEGAHIT v1.1.2
28D	ocean_biome	ocean	water	Monterey Bay, CA, USA	10/4/2016	36.835 N, 121.901 W	149.5	Metagenome	1174554	1174705	28D metaG	166761	https://genome.jgi.doe.gov/portal/28DmetaG_FD/28DmetaG_FD.download.html	467729	PRJNA467729	SRP155261	SAMN09201851	Gp0266613	Illumina HiSeq-2000	2 × 151	4,854,715,534	SPAdes assembler 3.11.1
28R	ocean_biome	ocean	water	Monterey Bay, CA, USA	10/4/2016	36.835 N, 121.901 W	149.5	Metatranscriptome	1174822	1174942	28R metaT	166842	https://genome.jgi.doe.gov/portal/28RmetaT_FD/28RmetaT_FD.download.html	502458	PRJNA502458	SRP174134	SAMN10350970	Gp0290792	Illumina HiSeq-2000	2 × 151	10,456,791,978	MEGAHIT v1.1.2
29D	ocean_biome	ocean	water	Monterey Bay, CA, USA	10/10/2016	36.835 N, 121.901 W	100	Metagenome	1174556	1174706	29D metaG	166762	https://genome.jgi.doe.gov/portal/29DmetaG_FD/29DmetaG_FD.download.html	467730	PRJNA467730	SRP155264	SAMN09202067	Gp0266614	Illumina HiSeq-2000	2 × 151	5,913,326,704	SPAdes assembler 3.11.1
29R	ocean_biome	ocean	water	Monterey Bay, CA, USA	10/10/2016	36.835 N, 121.901 W	100	Metatranscriptome	1174997	1175091	29R metaT	166883	https://genome.jgi.doe.gov/portal/29RmetaT_FD/29RmetaT_FD.download.html	468143	PRJNA468143	SRP155448	SAMN09201429	Gp0267189	Illumina HiSeq-2000	2 × 151	8,853,622,562	MEGAHIT v1.1.2
2D	ocean_biome	ocean	water	Monterey Bay, CA, USA	11/16/2016	36.835 N, 121.901 W	1000	Metagenome	1174506	1174681	2D metaG	166737	https://genome.jgi.doe.gov/portal/2DmetaG_FD/2DmetaG_FD.download.html	467721	PRJNA467721	SRP155237	SAMN09201419	Gp0266592	Illumina HiSeq-2000	2 × 151	7,983,910,278	SPAdes assembler 3.11.1
2R	ocean_biome	ocean	water	Monterey Bay, CA, USA	11/16/2016	36.835 N, 121.901 W	1000	Metatranscriptome	1174771	1174925	2R metaT	166825	https://genome.jgi.doe.gov/portal/2RmetaT_FD/2RmetaT_FD.download.html	468300	PRJNA468300	SRP155438	SAMN09202080	Gp0290775	Illumina HiSeq-2000	2 × 151	9,198,635,516	MEGAHIT v1.1.2
30D	ocean_biome	ocean	water	Monterey Bay, CA, USA	10/5/2016	36.835 N, 121.901 W	200	Metagenome	1174558	1174707	30D metaG	166763	https://genome.jgi.doe.gov/portal/30DmetaG_FD/30DmetaG_FD.download.html	502416	PRJNA502416	SRP174104	SAMN10350895	Gp0266615	Illumina HiSeq-2000	2 × 151	6,613,959,758	SPAdes assembler 3.11.1
30R	ocean_biome	ocean	water	Monterey Bay, CA, USA	10/5/2016	36.835 N, 121.901 W	200	Metatranscriptome	1174825	1174943	30R metaT	166843	https://genome.jgi.doe.gov/portal/30RmetaT_FD/30RmetaT_FD.download.html	468310	PRJNA468310	SRP163180	SAMN09201816	Gp0290793	Illumina HiSeq-2000	2 × 151	9,853,104,246	MEGAHIT v1.1.2
31D	ocean_biome	ocean	water	Monterey Bay, CA, USA	10/26/2016	36.835 N, 121.901 W	200	Metagenome	1174560	1174708	31D metaG	166764	https://genome.jgi.doe.gov/portal/31DmetaG_FD/31DmetaG_FD.download.html	467731	PRJNA467731	SRP155266	SAMN09201850	Gp0266616	Illumina HiSeq-2000	2 × 151	6,324,142,740	SPAdes assembler 3.11.1
31R	ocean_biome	ocean	water	Monterey Bay, CA, USA	10/26/2016	36.835 N, 121.901 W	200	Metatranscriptome	1175000	1175092	31R metaT	166884	https://genome.jgi.doe.gov/portal/31RmetaT_FD/31RmetaT_FD.download.html	467778	PRJNA467778	SRP155456	SAMN09201438	Gp0266673	Illumina HiSeq-2000	2 × 151	12,414,596,672	MEGAHIT v1.1.2
32D	ocean_biome	ocean	water	Monterey Bay, CA, USA	10/19/2016	36.835 N, 121.901 W	200	Metagenome	1174562	1174709	32D metaG	166765	https://genome.jgi.doe.gov/portal/32DmetaG_FD/32DmetaG_FD.download.html	468210	PRJNA468210	SRP155268	SAMN09201604	Gp0272199	Illumina HiSeq-2000	2 × 151	7,234,605,696	SPAdes assembler 3.11.1
32R	ocean_biome	ocean	water	Monterey Bay, CA, USA	10/19/2016	36.835 N, 121.901 W	200	Metatranscriptome	1175003	1175093	32R metaT	166885	https://genome.jgi.doe.gov/portal/32RmetaT_FD/32RmetaT_FD.download.html	468144	PRJNA468144	SRP155455	SAMN09202305	Gp0267190	Illumina HiSeq-2000	2 × 151	10,512,598,256	MEGAHIT v1.1.2
33D	ocean_biome	ocean	water	Monterey Bay, CA, USA	9/28/2016	36.835 N, 121.901 W	75	Metagenome	1174564	1174710	33D metaG	166766	https://genome.jgi.doe.gov/portal/33DmetaG_FD/33DmetaG_FD.download.html	502417	PRJNA502417	SRP174107	SAMN10351036	Gp0266617	Illumina HiSeq-2000	2 × 151	8,943,864,994	SPAdes assembler 3.11.1
33R	ocean_biome	ocean	water	Monterey Bay, CA, USA	9/28/2016	36.835 N, 121.901 W	75	Metatranscriptome	1174828	1174944	33R metaT	166844	https://genome.jgi.doe.gov/portal/33RmetaT_FD/33RmetaT_FD.download.html	502459	PRJNA502459	SRP174133	SAMN10350969	Gp0290794	Illumina HiSeq-2000	2 × 151	10,664,122,830	MEGAHIT v1.1.2
34D	ocean_biome	ocean	water	Monterey Bay, CA, USA	9/30/2016	36.835 N, 121.901 W	94	Metagenome	1174566	1174711	34D metaG	166767	https://genome.jgi.doe.gov/portal/34DmetaG_FD/34DmetaG_FD.download.html	467732	PRJNA467732	SRP155270	SAMN09201849	Gp0266618	Illumina HiSeq-2000	2 × 151	5,589,222,116	SPAdes assembler 3.11.1
34R	ocean_biome	ocean	water	Monterey Bay, CA, USA	9/30/2016	36.835 N, 121.901 W	94	Metatranscriptome	1174831	1174945	34R metaT	166845	https://genome.jgi.doe.gov/portal/34RmetaT_FD/34RmetaT_FD.download.html	468311	PRJNA468311	SRP163189	SAMN09202123	Gp0290795	Illumina HiSeq-2000	2 × 151	10,817,986,998	MEGAHIT v1.1.2
35D	ocean_biome	ocean	water	Monterey Bay, CA, USA	10/20/2016	36.835 N, 121.901 W	200	Metagenome	1174568	1174712	35D metaG	166768	https://genome.jgi.doe.gov/portal/35DmetaG_FD/35DmetaG_FD.download.html	502441	PRJNA502441	SRP174106	SAMN10351042	Gp0272200	Illumina HiSeq-2000	2 × 151	8,308,168,886	SPAdes assembler 3.11.1
35R	ocean_biome	ocean	water	Monterey Bay, CA, USA	10/20/2016	36.835 N, 121.901 W	200	Metatranscriptome	1175006	1175094	35R metaT	166886	https://genome.jgi.doe.gov/portal/35RmetaT_FD/35RmetaT_FD.download.html	467779	PRJNA467779	SRP155457	SAMN09201437	Gp0266674	Illumina HiSeq-2000	2 × 151	12,257,738,778	MEGAHIT v1.1.2
36D	ocean_biome	ocean	water	Monterey Bay, CA, USA	11/14/2016	36.835 N, 121.901 W	825	Metagenome	1174570	1174713	36D metaG	166769	https://genome.jgi.doe.gov/portal/36D_rmetaG_FD/36D_rmetaG_FD.download.html	467733	PRJNA467733	SRP155275	SAMN09202068	Gp0266619	Illumina HiSeq-2000	2 × 151	9,178,069,618	SPAdes assembler 3.11.1
36D_r	ocean_biome	ocean	water	Monterey Bay, CA, USA	10/5/2016	36.835 N, 121.901 W	200	Metagenome	1174672	1174764	36D_r metaG	166820	https://genome.jgi.doe.gov/portal/36DmetaG_FD/36DmetaG_FD.download.html	467770	PRJNA467770	SRP155426	SAMN09201533	Gp0266665	Illumina HiSeq-2000	2 × 151	5,877,069,792	SPAdes assembler 3.11.1
36R	ocean_biome	ocean	water	Monterey Bay, CA, USA	11/14/2016	36.835 N, 121.901 W	825	Metatranscriptome	1174834	1174946	36R metaT	166846	https://genome.jgi.doe.gov/portal/36R_rmetaT_FD/36R_rmetaT_FD.download.html	468312	PRJNA468312	SRP163183	SAMN09201815	Gp0290796	Illumina HiSeq-2000	2 × 151	9,511,697,776	MEGAHIT v1.1.2
36R_r	ocean_biome	ocean	water	Monterey Bay, CA, USA	10/5/2016	36.835 N, 121.901 W	200	Metatranscriptome	1175078	1175118	36R_r metaT	166910	https://genome.jgi.doe.gov/portal/36RmetaT_FD/36RmetaT_FD.download.html	468152	PRJNA468152	SRP155603	SAMN09201303	Gp0267198	Illumina HiSeq-2000	2 × 151	14,183,895,080	MEGAHIT v1.1.2
37D	ocean_biome	ocean	water	Monterey Bay, CA, USA	10/25/2016	36.835 N, 121.901 W	200	Metagenome	1174572	1174714	37D metaG	166770	https://genome.jgi.doe.gov/portal/37DmetaG_FD/37DmetaG_FD.download.html	467734	PRJNA467734	SRP155273	SAMN09202069	Gp0266620	Illumina HiSeq-2000	2 × 151	5,904,284,220	SPAdes assembler 3.11.1
38D	ocean_biome	ocean	water	Monterey Bay, CA, USA	10/2/2016	36.835 N, 121.901 W	175	Metagenome	1174574	1174715	38D metaG	166771	https://genome.jgi.doe.gov/portal/38DmetaG_FD/38DmetaG_FD.download.html	468211	PRJNA468211	SRP155280	SAMN09202469	Gp0272201	Illumina HiSeq-2000	2 × 151	7,854,968,358	SPAdes assembler 3.11.1
38R	ocean_biome	ocean	water	Monterey Bay, CA, USA	10/2/2016	36.835 N, 121.901 W	175	Metatranscriptome	1174837	1174947	38R metaT	166847	https://genome.jgi.doe.gov/portal/38RmetaT_FD/38RmetaT_FD.download.html	502460	PRJNA502460	SRP173009	SAMN10351182	Gp0290797	Illumina HiSeq-2000	2 × 151	9,291,317,504	MEGAHIT v1.1.2
39D	ocean_biome	ocean	water	Monterey Bay, CA, USA	9/28/2016	36.835 N, 121.901 W	75	Metagenome	1174576	1174716	39D metaG	166772	https://genome.jgi.doe.gov/portal/39DmetaG_FD/39DmetaG_FD.download.html	467735	PRJNA467735	SRP155282	SAMN09201451	Gp0266621	Illumina HiSeq-2000	2 × 151	9,772,488,366	SPAdes assembler 3.11.1
39R	ocean_biome	ocean	water	Monterey Bay, CA, USA	9/28/2016	36.835 N, 121.901 W	75	Metatranscriptome	1174840	1174948	39R metaT	166848	https://genome.jgi.doe.gov/portal/39RmetaT_FD/39RmetaT_FD.download.html	468313	PRJNA468313	SRP155441	SAMN09202124	Gp0290798	Illumina HiSeq-2000	2 × 151	9,537,885,102	MEGAHIT v1.1.2
3D	ocean_biome	ocean	water	Monterey Bay, CA, USA	11/9/2016	36.835 N, 121.901 W	175	Metagenome	1174508	1174682	3D metaG	166738	https://genome.jgi.doe.gov/portal/3DmetaG_FD/3DmetaG_FD.download.html	467722	PRJNA467722	SRP155238	SAMN09201454	Gp0266593	Illumina HiSeq-2000	2 × 151	6,667,059,814	SPAdes assembler 3.11.1
3R	ocean_biome	ocean	water	Monterey Bay, CA, USA	11/9/2016	36.835 N, 121.901 W	175	Metatranscriptome	1174774	1174926	3R metaT	166826	https://genome.jgi.doe.gov/portal/3RmetaT_FD/3RmetaT_FD.download.html	468301	PRJNA468301	SRP155439	SAMN09201821	Gp0290776	Illumina HiSeq-2000	2 × 151	9,116,905,256	MEGAHIT v1.1.2
40D	ocean_biome	ocean	water	Monterey Bay, CA, USA	11/4/2016	36.835 N, 121.901 W	400	Metagenome	1174578	1174717	40D metaG	166773	https://genome.jgi.doe.gov/portal/40DmetaG_FD/40DmetaG_FD.download.html	467736	PRJNA467736	SRP155287	SAMN09201848	Gp0266622	Illumina HiSeq-2000	2 × 151	8,607,890,598	SPAdes assembler 3.11.1
40R	ocean_biome	ocean	water	Monterey Bay, CA, USA	11/4/2016	36.835 N, 121.901 W	400	Metatranscriptome	1174843	1174949	40R metaT	166849	https://genome.jgi.doe.gov/portal/40RmetaT_FD/40RmetaT_FD.download.html	468314	PRJNA468314	SRP163274	SAMN09201262	Gp0290799	Illumina HiSeq-2000	2 × 151	8,161,434,334	MEGAHIT v1.1.2
41D	ocean_biome	ocean	water	Monterey Bay, CA, USA	11/13/2016	36.835 N, 121.901 W	350	Metagenome	1174580	1174718	41D metaG	166774	https://genome.jgi.doe.gov/portal/41DmetaG_FD/41DmetaG_FD.download.html	467737	PRJNA467737	SRP155292	SAMN09201681	Gp0266623	Illumina HiSeq-2000	2 × 151	7,636,600,916	SPAdes assembler 3.11.1
41R	ocean_biome	ocean	water	Monterey Bay, CA, USA	11/13/2016	36.835 N, 121.901 W	350	Metatranscriptome	1174846	1174950	41R metaT	166850	https://genome.jgi.doe.gov/portal/41RmetaT_FD/41RmetaT_FD.download.html	502461	PRJNA502461	SRP174136	SAMN10351183	Gp0290800	Illumina HiSeq-2000	2 × 151	10,445,115,752	MEGAHIT v1.1.2
42D	ocean_biome	ocean	water	Monterey Bay, CA, USA	9/27/2016	36.835 N, 121.901 W	75	Metagenome	1174582	1174719	42D metaG	166775	https://genome.jgi.doe.gov/portal/42DmetaG_FD/42DmetaG_FD.download.html	502418	PRJNA502418	SRP174105	SAMN10350894	Gp0266624	Illumina HiSeq-2000	2 × 151	9,179,809,138	SPAdes assembler 3.11.1
42R	ocean_biome	ocean	water	Monterey Bay, CA, USA	9/27/2016	36.835 N, 121.901 W	75	Metatranscriptome	1174849	1174951	42R metaT	166851	https://genome.jgi.doe.gov/portal/42RmetaT_FD/42RmetaT_FD.download.html	502462	PRJNA502462	SRP173010	SAMN10350968	Gp0290801	Illumina HiSeq-2000	2 × 151	10,001,448,458	MEGAHIT v1.1.2
43D	ocean_biome	ocean	water	Monterey Bay, CA, USA	11/8/2016	36.835 N, 121.901 W	225	Metagenome	1174584	1174720	43D metaG	166776	https://genome.jgi.doe.gov/portal/43DmetaG_FD/43DmetaG_FD.download.html	468212	PRJNA468212	SRP155294	SAMN09201603	Gp0272202	Illumina HiSeq-2000	2 × 151	7,671,333,030	SPAdes assembler 3.11.1
43R	ocean_biome	ocean	water	Monterey Bay, CA, USA	11/8/2016	36.835 N, 121.901 W	225	Metatranscriptome	1174852	1174952	43R metaT	166852	https://genome.jgi.doe.gov/portal/43RmetaT_FD/43RmetaT_FD.download.html	502463	PRJNA502463	SRP174135	SAMN10350967	Gp0290802	Illumina HiSeq-2000	2 × 151	10,303,982,092	MEGAHIT v1.1.2
44D	ocean_biome	ocean	water	Monterey Bay, CA, USA	9/30/2016	36.835 N, 121.901 W	94.1	Metagenome	1174586	1174721	44D metaG	166777	https://genome.jgi.doe.gov/portal/44DmetaG_FD/44DmetaG_FD.download.html	467738	PRJNA467738	SRP155297	SAMN09201847	Gp0266625	Illumina HiSeq-2000	2 × 151	8,361,881,096	SPAdes assembler 3.11.1
44R	ocean_biome	ocean	water	Monterey Bay, CA, USA	9/30/2016	36.835 N, 121.901 W	94.1	Metatranscriptome	1174855	1174953	44R metaT	166853	https://genome.jgi.doe.gov/portal/44RmetaT_FD/44RmetaT_FD.download.html	468315	PRJNA468315	SRP163282	SAMN09202125	Gp0290803	Illumina HiSeq-2000	2 × 151	8,829,029,192	MEGAHIT v1.1.2
45D	ocean_biome	ocean	water	Monterey Bay, CA, USA	11/3/2016	36.835 N, 121.901 W	300	Metagenome	1174588	1174722	45D metaG	166778	https://genome.jgi.doe.gov/portal/45DmetaG_FD/45DmetaG_FD.download.html	467739	PRJNA467739	SRP155298	SAMN09201682	Gp0266626	Illumina HiSeq-2000	2 × 151	6,945,518,612	SPAdes assembler 3.11.1
45R	ocean_biome	ocean	water	Monterey Bay, CA, USA	11/3/2016	36.835 N, 121.901 W	300	Metatranscriptome	1174858	1174954	45R metaT	166854	https://genome.jgi.doe.gov/portal/45RmetaT_FD/45RmetaT_FD.download.html	502464	PRJNA502464	SRP174137	SAMN10351124	Gp0290804	Illumina HiSeq-2000	2 × 151	9,459,974,538	MEGAHIT v1.1.2
46D	ocean_biome	ocean	water	Monterey Bay, CA, USA	10/21/2016	36.835 N, 121.901 W	200	Metagenome	1174590	1174723	46D metaG	166779	https://genome.jgi.doe.gov/portal/46DmetaG_FD/46DmetaG_FD.download.html	468213	PRJNA468213	SRP155299	SAMN09201602	Gp0272203	Illumina HiSeq-2000	2 × 151	7,398,835,108	SPAdes assembler 3.11.1
46R	ocean_biome	ocean	water	Monterey Bay, CA, USA	10/21/2016	36.835 N, 121.901 W	200	Metatranscriptome	1174861	1174955	46R metaT	166855	https://genome.jgi.doe.gov/portal/46RmetaT_FD/46RmetaT_FD.download.html	468316	PRJNA468316	SRP163285	SAMN09202126	Gp0290805	Illumina HiSeq-2000	2 × 151	9,167,903,090	MEGAHIT v1.1.2
47D	ocean_biome	ocean	water	Monterey Bay, CA, USA	10/25/2016	36.835 N, 121.901 W	200	Metagenome	1174592	1174724	47D metaG	166780	https://genome.jgi.doe.gov/portal/47DmetaG_FD/47DmetaG_FD.download.html	467740	PRJNA467740	SRP155307	SAMN09201846	Gp0266627	Illumina HiSeq-2000	2 × 151	7,252,908,708	SPAdes assembler 3.11.1
47R	ocean_biome	ocean	water	Monterey Bay, CA, USA	10/25/2016	36.835 N, 121.901 W	200	Metatranscriptome	1175012	1175096	47R metaT	166888	https://genome.jgi.doe.gov/portal/47RmetaT_FD/47RmetaT_FD.download.html	467780	PRJNA467780	SRP155461	SAMN09201301	Gp0266675	Illumina HiSeq-2000	2 × 151	11,208,036,608	MEGAHIT v1.1.2
48D	ocean_biome	ocean	water	Monterey Bay, CA, USA	10/31/2016	36.835 N, 121.901 W	300	Metagenome	1174594	1174725	48D metaG	166781	https://genome.jgi.doe.gov/portal/48D_rmetaG_FD/48D_rmetaG_FD.download.html	467741	PRJNA467741	SRP155306	SAMN09201450	Gp0266628	Illumina HiSeq-2000	2 × 151	7,977,827,394	SPAdes assembler 3.11.1
48D_r	ocean_biome	ocean	water	Monterey Bay, CA, USA	11/2/2016	36.835 N, 121.901 W	550	Metagenome	1174674	1174765	48D_r metaG	166821	https://genome.jgi.doe.gov/portal/48DmetaG_FD/48DmetaG_FD.download.html	467771	PRJNA467771	SRP155431	SAMN09201532	Gp0266666	Illumina HiSeq-2000	2 × 151	8,114,732,752	SPAdes assembler 3.11.1
48R	ocean_biome	ocean	water	Monterey Bay, CA, USA	10/31/2016	36.835 N, 121.901 W	300	Metatranscriptome	1174864	1174956	48R metaT	166856	https://genome.jgi.doe.gov/portal/48RmetaT_FD/48RmetaT_FD.download.html	468317	PRJNA468317	SRP163284	SAMN09201261	Gp0290806	Illumina HiSeq-2000	2 × 151	9,928,027,426	MEGAHIT v1.1.2
49D	ocean_biome	ocean	water	Monterey Bay, CA, USA	10/3/2016	36.835 N, 121.901 W	325	Metagenome	1174596	1174726	49D metaG	166782	https://genome.jgi.doe.gov/portal/49DmetaG_FD/49DmetaG_FD.download.html	502442	PRJNA502442	SRP174109	SAMN10351356	Gp0272204	Illumina HiSeq-2000	2 × 151	8,952,643,228	SPAdes assembler 3.11.1
49R	ocean_biome	ocean	water	Monterey Bay, CA, USA	10/3/2016	36.835 N, 121.901 W	325	Metatranscriptome	1174867	1174957	49R metaT	166857	https://genome.jgi.doe.gov/portal/49RmetaT_FD/49RmetaT_FD.download.html	468318	PRJNA468318	SRP163276	SAMN09201260	Gp0290807	Illumina HiSeq-2000	2 × 151	8,642,543,890	MEGAHIT v1.1.2
4D	ocean_biome	ocean	water	Monterey Bay, CA, USA	11/3/2016	36.835 N, 121.901 W	325	Metagenome	1174510	1174683	4D metaG	166739	https://genome.jgi.doe.gov/portal/4DmetaG_FD/4DmetaG_FD.download.html	467723	PRJNA467723	SRP155240	SAMN09201857	Gp0266594	Illumina HiSeq-2000	2 × 151	7,717,664,360	SPAdes assembler 3.11.1
4R	ocean_biome	ocean	water	Monterey Bay, CA, USA	11/3/2016	36.835 N, 121.901 W	325	Metatranscriptome	1174777	1174927	4R metaT	166827	https://genome.jgi.doe.gov/portal/4RmetaT_FD/4RmetaT_FD.download.html	468302	PRJNA468302	SRP161170	SAMN09201263	Gp0290777	Illumina HiSeq-2000	2 × 151	10,907,101,460	MEGAHIT v1.1.2
50D	ocean_biome	ocean	water	Monterey Bay, CA, USA	10/18/2016	36.835 N, 121.901 W	200	Metagenome	1174598	1174727	50D metaG	166783	https://genome.jgi.doe.gov/portal/50DmetaG_FD/50DmetaG_FD.download.html	502419	PRJNA502419	SRP174111	SAMN10351174	Gp0266629	Illumina HiSeq-2000	2 × 151	7,899,520,304	SPAdes assembler 3.11.1
50R	ocean_biome	ocean	water	Monterey Bay, CA, USA	10/18/2016	36.835 N, 121.901 W	200	Metatranscriptome	1175015	1175097	50R metaT	166889	https://genome.jgi.doe.gov/portal/50RmetaT_FD/50RmetaT_FD.download.html	467781	PRJNA467781	SRP155462	SAMN09201714	Gp0266676	Illumina HiSeq-2000	2 × 151	11,745,765,426	MEGAHIT v1.1.2
51D	ocean_biome	ocean	water	Monterey Bay, CA, USA	11/13/2016	36.835 N, 121.901 W	400	Metagenome	1174600	1174728	51D metaG	166784	https://genome.jgi.doe.gov/portal/51DmetaG_FD/51DmetaG_FD.download.html	502420	PRJNA502420	SRP174112	SAMN10351052	Gp0266630	Illumina HiSeq-2000	2 × 151	6,003,670,004	SPAdes assembler 3.11.1
51R	ocean_biome	ocean	water	Monterey Bay, CA, USA	11/13/2016	36.835 N, 121.901 W	400	Metatranscriptome	1174870	1174958	51R metaT	166858	https://genome.jgi.doe.gov/portal/51RmetaT_FD/51RmetaT_FD.download.html	468319	PRJNA468319	SRP163283	SAMN09202127	Gp0290808	Illumina HiSeq-2000	2 × 151	11,738,448,570	MEGAHIT v1.1.2
52D	ocean_biome	ocean	water	Monterey Bay, CA, USA	10/17/2016	36.835 N, 121.901 W	200	Metagenome	1174602	1174729	52D metaG	166785	https://genome.jgi.doe.gov/portal/52DmetaG_FD/52DmetaG_FD.download.html	467742	PRJNA467742	SRP155308	SAMN09201449	Gp0266631	Illumina HiSeq-2000	2 × 151	5,968,743,100	SPAdes assembler 3.11.1
52R	ocean_biome	ocean	water	Monterey Bay, CA, USA	10/17/2016	36.835 N, 121.901 W	200	Metatranscriptome	1175018	1175098	52R metaT	166890	https://genome.jgi.doe.gov/portal/52RmetaT_FD/52RmetaT_FD.download.html	467782	PRJNA467782	SRP155463	SAMN09202118	Gp0266677	Illumina HiSeq-2000	2 × 151	11,715,640,926	MEGAHIT v1.1.2
53D	ocean_biome	ocean	water	Monterey Bay, CA, USA	9/29/2016	36.835 N, 121.901 W	125	Metagenome	1174604	1174730	53D metaG	166786	https://genome.jgi.doe.gov/portal/53DmetaG_FD/53DmetaG_FD.download.html	502421	PRJNA502421	SRP174114	SAMN10351091	Gp0266632	Illumina HiSeq-2000	2 × 151	10,237,618,498	SPAdes assembler 3.11.1
53R	ocean_biome	ocean	water	Monterey Bay, CA, USA	9/29/2016	36.835 N, 121.901 W	125	Metatranscriptome	1174873	1174959	53R metaT	166859	https://genome.jgi.doe.gov/portal/53RmetaT_FD/53RmetaT_FD.download.html	468320	PRJNA468320	SRP163278	SAMN09201259	Gp0290809	Illumina HiSeq-2000	2 × 151	8,487,681,914	MEGAHIT v1.1.2
54D	ocean_biome	ocean	water	Monterey Bay, CA, USA	11/11/2016	36.835 N, 121.901 W	425	Metagenome	1174606	1174731	54D metaG	166787	https://genome.jgi.doe.gov/portal/54DmetaG_FD/54DmetaG_FD.download.html	468214	PRJNA468214	SRP155314	SAMN09202493	Gp0272205	Illumina HiSeq-2000	2 × 151	8,189,482,886	SPAdes assembler 3.11.1
54R	ocean_biome	ocean	water	Monterey Bay, CA, USA	11/11/2016	36.835 N, 121.901 W	425	Metatranscriptome	1174876	1174960	54R metaT	166860	https://genome.jgi.doe.gov/portal/54RmetaT_FD/54RmetaT_FD.download.html	468321	PRJNA468321	SRP163277	SAMN09201279	Gp0290810	Illumina HiSeq-2000	2 × 151	10,432,362,896	MEGAHIT v1.1.2
55D	ocean_biome	ocean	water	Monterey Bay, CA, USA	11/8/2016	36.835 N, 121.901 W	175	Metagenome	1174608	1174732	55D metaG	166788	https://genome.jgi.doe.gov/portal/55DmetaG_FD/55DmetaG_FD.download.html	502422	PRJNA502422	SRP174115	SAMN10351092	Gp0266633	Illumina HiSeq-2000	2 × 151	9,803,128,682	SPAdes assembler 3.11.1
55R	ocean_biome	ocean	water	Monterey Bay, CA, USA	11/8/2016	36.835 N, 121.901 W	175	Metatranscriptome	1175021	1175099	55R metaT	166891	https://genome.jgi.doe.gov/portal/55RmetaT_FD/55RmetaT_FD.download.html	468145	PRJNA468145	SRP155489	SAMN09201664	Gp0267191	Illumina HiSeq-2000	2 × 151	11,401,846,316	MEGAHIT v1.1.2
56D	ocean_biome	ocean	water	Monterey Bay, CA, USA	11/9/2016	36.835 N, 121.901 W	174.4	Metagenome	1174610	1174733	56D metaG	166789	https://genome.jgi.doe.gov/portal/56DmetaG_FD/56DmetaG_FD.download.html	467743	PRJNA467743	SRP155320	SAMN09201683	Gp0266634	Illumina HiSeq-2000	2 × 151	4,883,065,180	SPAdes assembler 3.11.1
56R	ocean_biome	ocean	water	Monterey Bay, CA, USA	11/9/2016	36.835 N, 121.901 W	174.4	Metatranscriptome	1174879	1174961	56R metaT	166861	https://genome.jgi.doe.gov/portal/56RmetaT_FD/56RmetaT_FD.download.html	468322	PRJNA468322	SRP163275	SAMN09202128	Gp0290811	Illumina HiSeq-2000	2 × 151	9,136,023,970	MEGAHIT v1.1.2
57D	ocean_biome	ocean	water	Monterey Bay, CA, USA	11/6/2016	36.835 N, 121.901 W	550	Metagenome	1174612	1174734	57D metaG	166790	https://genome.jgi.doe.gov/portal/57DmetaG_FD/57DmetaG_FD.download.html	502423	PRJNA502423	SRP174120	SAMN10351051	Gp0266635	Illumina HiSeq-2000	2 × 151	8,240,164,526	SPAdes assembler 3.11.1
57R	ocean_biome	ocean	water	Monterey Bay, CA, USA	11/6/2016	36.835 N, 121.901 W	550	Metatranscriptome	1174882	1174962	57R metaT	166862	https://genome.jgi.doe.gov/portal/57RmetaT_FD/57RmetaT_FD.download.html	468323	PRJNA468323	SRP163281	SAMN09201278	Gp0290812	Illumina HiSeq-2000	2 × 151	10,912,252,070	MEGAHIT v1.1.2
58D	ocean_biome	ocean	water	Monterey Bay, CA, USA	11/4/2016	36.835 N, 121.901 W	400	Metagenome	1174614	1174735	58D metaG	166791	https://genome.jgi.doe.gov/portal/58D_rmetaG_FD/58D_rmetaG_FD.download.html	502424	PRJNA502424	SRP174121	SAMN10351093	Gp0266636	Illumina HiSeq-2000	2 × 151	10,007,475,472	SPAdes assembler 3.11.1
58D_r	ocean_biome	ocean	water	Monterey Bay, CA, USA	11/4/2016	36.835 N, 121.901 W	425	Metagenome	1174676	1174766	58D_r metaG	166822	https://genome.jgi.doe.gov/portal/58DmetaG_FD/58DmetaG_FD.download.html	467772	PRJNA467772	SRP155434	SAMN09202114	Gp0266667	Illumina HiSeq-2000	2 × 151	7,642,683,196	SPAdes assembler 3.11.1
58R	ocean_biome	ocean	water	Monterey Bay, CA, USA	11/4/2016	36.835 N, 121.901 W	400	Metatranscriptome	1175024	1175100	58R metaT	166892	https://genome.jgi.doe.gov/portal/58R_rmetaT_FD/58R_rmetaT_FD.download.html	467783	PRJNA467783	SRP155488	SAMN09201436	Gp0266678	Illumina HiSeq-2000	2 × 151	11,675,230,004	MEGAHIT v1.1.2
58R_r	ocean_biome	ocean	water	Monterey Bay, CA, USA	11/4/2016	36.835 N, 121.901 W	425	Metatranscriptome	1174918	1174974	58R_r metaT	166874	https://genome.jgi.doe.gov/portal/58RmetaT_FD/58RmetaT_FD.download.html	468331	PRJNA468331	SRP163295	SAMN09200098	Gp0290824	Illumina HiSeq-2000	2 × 151	7,024,482,250	MEGAHIT v1.1.2
59D	ocean_biome	ocean	water	Monterey Bay, CA, USA	10/21/2016	36.835 N, 121.901 W	200	Metagenome	1174616	1174736	59D metaG	166792	https://genome.jgi.doe.gov/portal/59DmetaG_FD/59DmetaG_FD.download.html	467744	PRJNA467744	SRP155321	SAMN09201444	Gp0266637	Illumina HiSeq-2000	2 × 151	8,505,201,538	SPAdes assembler 3.11.1
60D	ocean_biome	ocean	water	Monterey Bay, CA, USA	11/12/2016	36.835 N, 121.901 W	475	Metagenome	1174618	1174737	60D metaG	166793	https://genome.jgi.doe.gov/portal/60DmetaG_FD/60DmetaG_FD.download.html	502425	PRJNA502425	SRP174119	SAMN10351050	Gp0266638	Illumina HiSeq-2000	2 × 151	7,531,245,498	SPAdes assembler 3.11.1
60R	ocean_biome	ocean	water	Monterey Bay, CA, USA	11/12/2016	36.835 N, 121.901 W	475	Metatranscriptome	1174885	1174963	60R metaT	166863	https://genome.jgi.doe.gov/portal/60RmetaT_FD/60RmetaT_FD.download.html	502465	PRJNA502465	SRP173012	SAMN10351125	Gp0290813	Illumina HiSeq-2000	2 × 151	9,036,901,228	MEGAHIT v1.1.2
61D	ocean_biome	ocean	water	Monterey Bay, CA, USA	10/31/2016	36.835 N, 121.901 W	290	Metagenome	1174620	1174738	61D metaG	166794	https://genome.jgi.doe.gov/portal/61DmetaG_FD/61DmetaG_FD.download.html	502426	PRJNA502426	SRP174118	SAMN10351049	Gp0266639	Illumina HiSeq-2000	2 × 151	8,327,099,756	SPAdes assembler 3.11.1
61R	ocean_biome	ocean	water	Monterey Bay, CA, USA	10/31/2016	36.835 N, 121.901 W	290	Metatranscriptome	1175030	1175102	61R metaT	166894	https://genome.jgi.doe.gov/portal/61RmetaT_FD/61RmetaT_FD.download.html	468146	PRJNA468146	SRP155487	SAMN09201304	Gp0267192	Illumina HiSeq-2000	2 × 151	12,760,280,972	MEGAHIT v1.1.2
62D	ocean_biome	ocean	water	Monterey Bay, CA, USA	10/28/2016	36.835 N, 121.901 W	250	Metagenome	1174622	1174739	62D metaG	166795	https://genome.jgi.doe.gov/portal/62DmetaG_FD/62DmetaG_FD.download.html	467745	PRJNA467745	SRP155334	SAMN09201274	Gp0266640	Illumina HiSeq-2000	2 × 151	7,880,205,894	SPAdes assembler 3.11.1
63D	ocean_biome	ocean	water	Monterey Bay, CA, USA	10/24/2016	36.835 N, 121.901 W	250	Metagenome	1174624	1174740	63D metaG	166796	https://genome.jgi.doe.gov/portal/63DmetaG_FD/63DmetaG_FD.download.html	467746	PRJNA467746	SRP155339	SAMN09202070	Gp0266641	Illumina HiSeq-2000	2 × 151	8,133,342,596	SPAdes assembler 3.11.1
63R	ocean_biome	ocean	water	Monterey Bay, CA, USA	10/24/2016	36.835 N, 121.901 W	250	Metatranscriptome	1175036	1175104	63R metaT	166896	https://genome.jgi.doe.gov/portal/63RmetaT_FD/63RmetaT_FD.download.html	468147	PRJNA468147	SRP155506	SAMN09202403	Gp0267193	Illumina HiSeq-2000	2 × 151	10,302,220,526	MEGAHIT v1.1.2
64D	ocean_biome	ocean	water	Monterey Bay, CA, USA	10/5/2016	36.835 N, 121.901 W	199.2	Metagenome	1174626	1174741	64D metaG	166797	https://genome.jgi.doe.gov/portal/64DmetaG_FD/64DmetaG_FD.download.html	467747	PRJNA467747	SRP155340	SAMN09202071	Gp0266642	Illumina HiSeq-2000	2 × 151	8,079,854,168	SPAdes assembler 3.11.1
65D	ocean_biome	ocean	water	Monterey Bay, CA, USA	11/2/2016	36.835 N, 121.901 W	400	Metagenome	1174628	1174742	65D metaG	166798	https://genome.jgi.doe.gov/portal/65DmetaG_FD/65DmetaG_FD.download.html	467748	PRJNA467748	SRP155355	SAMN09201541	Gp0266643	Illumina HiSeq-2000	2 × 151	6,290,782,310	SPAdes assembler 3.11.1
66D	ocean_biome	ocean	water	Monterey Bay, CA, USA	10/3/2016	36.835 N, 121.901 W	250	Metagenome	1174630	1174743	66D metaG	166799	https://genome.jgi.doe.gov/portal/66DmetaG_FD/66DmetaG_FD.download.html	467749	PRJNA467749	SRP155353	SAMN09202072	Gp0266644	Illumina HiSeq-2000	2 × 151	4,930,952,112	SPAdes assembler 3.11.1
66R	ocean_biome	ocean	water	Monterey Bay, CA, USA	10/3/2016	36.835 N, 121.901 W	250	Metatranscriptome	1174888	1174964	66R metaT	166864	https://genome.jgi.doe.gov/portal/66RmetaT_FD/66RmetaT_FD.download.html	502466	PRJNA502466	SRP174138	SAMN10350916	Gp0290814	Illumina HiSeq-2000	2 × 151	10,330,806,336	MEGAHIT v1.1.2
67D	ocean_biome	ocean	water	Monterey Bay, CA, USA	11/15/2016	36.835 N, 121.901 W	1000	Metagenome	1174632	1174744	67D metaG	166800	https://genome.jgi.doe.gov/portal/67DmetaG_FD/67DmetaG_FD.download.html	467750	PRJNA467750	SRP155356	SAMN09201540	Gp0266645	Illumina HiSeq-2000	2 × 151	6,280,811,478	SPAdes assembler 3.11.1
67R	ocean_biome	ocean	water	Monterey Bay, CA, USA	11/15/2016	36.835 N, 121.901 W	1000	Metatranscriptome	1175045	1175107	67R metaT	166899	https://genome.jgi.doe.gov/portal/67RmetaT_FD/67RmetaT_FD.download.html	468148	PRJNA468148	SRP155500	SAMN09201529	Gp0267194	Illumina HiSeq-2000	2 × 151	10,617,839,518	MEGAHIT v1.1.2
68D	ocean_biome	ocean	water	Monterey Bay, CA, USA	11/7/2016	36.835 N, 121.901 W	575	Metagenome	1174634	1174745	68D metaG	166801	https://genome.jgi.doe.gov/portal/68DmetaG_FD/68DmetaG_FD.download.html	467751	PRJNA467751	SRP155364	SAMN09202073	Gp0266646	Illumina HiSeq-2000	2 × 151	5,858,217,744	SPAdes assembler 3.11.1
68R	ocean_biome	ocean	water	Monterey Bay, CA, USA	11/7/2016	36.835 N, 121.901 W	575	Metatranscriptome	1174891	1174965	68R metaT	166865	https://genome.jgi.doe.gov/portal/68RmetaT_FD/68RmetaT_FD.download.html	502467	PRJNA502467	SRP173013	SAMN10350915	Gp0290815	Illumina HiSeq-2000	2 × 151	9,554,211,222	MEGAHIT v1.1.2
70D	ocean_biome	ocean	water	Monterey Bay, CA, USA	10/7/2016	36.835 N, 121.901 W	207.5	Metagenome	1174636	1174746	70D metaG	166802	https://genome.jgi.doe.gov/portal/70DmetaG_FD/70DmetaG_FD.download.html	467752	PRJNA467752	SRP155365	SAMN09201539	Gp0266647	Illumina HiSeq-2000	2 × 151	6,470,832,294	SPAdes assembler 3.11.1
70R	ocean_biome	ocean	water	Monterey Bay, CA, USA	10/7/2016	36.835 N, 121.901 W	207.5	Metatranscriptome	1174894	1174966	70R metaT	166866	https://genome.jgi.doe.gov/portal/70RmetaT_FD/70RmetaT_FD.download.html	468324	PRJNA468324	SRP163290	SAMN09202129	Gp0290816	Illumina HiSeq-2000	2 × 151	10,602,724,418	MEGAHIT v1.1.2
71D	ocean_biome	ocean	water	Monterey Bay, CA, USA	10/18/2016	36.835 N, 121.901 W	200	Metagenome	1174638	1174747	71D metaG	166803	https://genome.jgi.doe.gov/portal/71DmetaG_FD/71DmetaG_FD.download.html	467753	PRJNA467753	SRP155366	SAMN09201443	Gp0266648	Illumina HiSeq-2000	2 × 151	8,687,854,460	SPAdes assembler 3.11.1
71R	ocean_biome	ocean	water	Monterey Bay, CA, USA	10/18/2016	36.835 N, 121.901 W	200	Metatranscriptome	1175048	1175108	71R metaT	166900	https://genome.jgi.doe.gov/portal/71RmetaT_FD/71RmetaT_FD.download.html	467784	PRJNA467784	SRP155502	SAMN09202119	Gp0266682	Illumina HiSeq-2000	2 × 151	12,304,237,416	MEGAHIT v1.1.2
73D	ocean_biome	ocean	water	Monterey Bay, CA, USA	11/16/2016	36.835 N, 121.901 W	1000	Metagenome	1174640	1174748	73D metaG	166804	https://genome.jgi.doe.gov/portal/73DmetaG_FD/73DmetaG_FD.download.html	467754	PRJNA467754	SRP155376	SAMN09202074	Gp0266649	Illumina HiSeq-2000	2 × 151	6,269,792,102	SPAdes assembler 3.11.1
73R	ocean_biome	ocean	water	Monterey Bay, CA, USA	11/16/2016	36.835 N, 121.901 W	1000	Metatranscriptome	1174897	1174967	73R metaT	166867	https://genome.jgi.doe.gov/portal/73RmetaT_FD/73RmetaT_FD.download.html	468325	PRJNA468325	SRP163289	SAMN09201277	Gp0290817	Illumina HiSeq-2000	2 × 151	8,528,379,736	MEGAHIT v1.1.2
74D	ocean_biome	ocean	water	Monterey Bay, CA, USA	11/11/2016	36.835 N, 121.901 W	425	Metagenome	1174642	1174749	74D metaG	166805	https://genome.jgi.doe.gov/portal/74DmetaG_FD/74DmetaG_FD.download.html	467755	PRJNA467755	SRP155377	SAMN09201442	Gp0266650	Illumina HiSeq-2000	2 × 151	7,965,995,034	SPAdes assembler 3.11.1
75D	ocean_biome	ocean	water	Monterey Bay, CA, USA	11/14/2016	36.835 N, 121.901 W	825	Metagenome	1174644	1174750	75D metaG	166806	https://genome.jgi.doe.gov/portal/75DmetaG_FD/75DmetaG_FD.download.html	467756	PRJNA467756	SRP155385	SAMN09201538	Gp0266651	Illumina HiSeq-2000	2 × 151	5,308,517,646	SPAdes assembler 3.11.1
75R	ocean_biome	ocean	water	Monterey Bay, CA, USA	11/14/2016	36.835 N, 121.901 W	825	Metatranscriptome	1174900	1174968	75R metaT	166868	https://genome.jgi.doe.gov/portal/75RmetaT_FD/75RmetaT_FD.download.html	468326	PRJNA468326	SRP163291	SAMN09201276	Gp0290818	Illumina HiSeq-2000	2 × 151	9,110,384,170	MEGAHIT v1.1.2
76D	ocean_biome	ocean	water	Monterey Bay, CA, USA	11/10/2016	36.835 N, 121.901 W	350	Metagenome	1174646	1174751	76D metaG	166807	https://genome.jgi.doe.gov/portal/76DmetaG_FD/76DmetaG_FD.download.html	467757	PRJNA467757	SRP155391	SAMN09201302	Gp0266652	Illumina HiSeq-2000	2 × 151	7,179,999,868	SPAdes assembler 3.11.1
76R	ocean_biome	ocean	water	Monterey Bay, CA, USA	11/10/2016	36.835 N, 121.901 W	350	Metatranscriptome	1174903	1174969	76R metaT	166869	https://genome.jgi.doe.gov/portal/76RmetaT_FD/76RmetaT_FD.download.html	502468	PRJNA502468	SRP173016	SAMN10351184	Gp0290819	Illumina HiSeq-2000	2 × 151	8,145,636,412	MEGAHIT v1.1.2
77D	ocean_biome	ocean	water	Monterey Bay, CA, USA	10/7/2016	36.835 N, 121.901 W	201.5	Metagenome	1174648	1174752	77D metaG	166808	https://genome.jgi.doe.gov/portal/77DmetaG_FD/77DmetaG_FD.download.html	467758	PRJNA467758	SRP155398	SAMN09201684	Gp0266653	Illumina HiSeq-2000	2 × 151	6,424,234,902	SPAdes assembler 3.11.1
77R	ocean_biome	ocean	water	Monterey Bay, CA, USA	10/7/2016	36.835 N, 121.901 W	201.5	Metatranscriptome	1174906	1174970	77R metaT	166870	https://genome.jgi.doe.gov/portal/77RmetaT_FD/77RmetaT_FD.download.html	468327	PRJNA468327	SRP163294	SAMN09202166	Gp0290820	Illumina HiSeq-2000	2 × 151	11,267,917,772	MEGAHIT v1.1.2
78D	ocean_biome	ocean	water	Monterey Bay, CA, USA	9/26/2016	36.835 N, 121.901 W	92.5	Metagenome	1174650	1174753	78D metaG	166809	https://genome.jgi.doe.gov/portal/78DmetaG_FD/78DmetaG_FD.download.html	467759	PRJNA467759	SRP155399	SAMN09201441	Gp0266654	Illumina HiSeq-2000	2 × 151	7,805,644,812	SPAdes assembler 3.11.1
78R	ocean_biome	ocean	water	Monterey Bay, CA, USA	9/26/2016	36.835 N, 121.901 W	92.5	Metatranscriptome	1175054	1175110	78R metaT	166902	https://genome.jgi.doe.gov/portal/78RmetaT_FD/78RmetaT_FD.download.html	468149	PRJNA468149	SRP155516	SAMN09202306	Gp0267195	Illumina HiSeq-2000	2 × 151	10,213,209,650	MEGAHIT v1.1.2
79D	ocean_biome	ocean	water	Monterey Bay, CA, USA	11/7/2016	36.835 N, 121.901 W	550	Metagenome	1174652	1174754	79D metaG	166810	https://genome.jgi.doe.gov/portal/79DmetaG_FD/79DmetaG_FD.download.html	467760	PRJNA467760	SRP155406	SAMN09201685	Gp0266655	Illumina HiSeq-2000	2 × 151	5,990,380,192	SPAdes assembler 3.11.1
79R	ocean_biome	ocean	water	Monterey Bay, CA, USA	11/7/2016	36.835 N, 121.901 W	550	Metatranscriptome	1174909	1174971	79R metaT	166871	https://genome.jgi.doe.gov/portal/79RmetaT_FD/79RmetaT_FD.download.html	468328	PRJNA468328	SRP163293	SAMN09201275	Gp0290821	Illumina HiSeq-2000	2 × 151	8,943,879,490	MEGAHIT v1.1.2
7D	ocean_biome	ocean	water	Monterey Bay, CA, USA	10/26/2016	36.835 N, 121.901 W	200	Metagenome	1174514	1174685	7D metaG	166741	https://genome.jgi.doe.gov/portal/7DmetaG_FD/7DmetaG_FD.download.html	502407	PRJNA502407	SRP174087	SAMN10351037	Gp0266596	Illumina HiSeq-2000	2 × 151	10,931,783,316	SPAdes assembler 3.11.1
80D	ocean_biome	ocean	water	Monterey Bay, CA, USA	10/11/2016	36.835 N, 121.901 W	100	Metagenome	1174654	1174755	80D metaG	166811	https://genome.jgi.doe.gov/portal/80DmetaG_FD/80DmetaG_FD.download.html	467761	PRJNA467761	SRP155408	SAMN09201537	Gp0266656	Illumina HiSeq-2000	2 × 151	6,844,779,566	SPAdes assembler 3.11.1
80R	ocean_biome	ocean	water	Monterey Bay, CA, USA	10/11/2016	36.835 N, 121.901 W	100	Metatranscriptome	1175057	1175111	80R metaT	166903	https://genome.jgi.doe.gov/portal/80RmetaT_FD/80RmetaT_FD.download.html	467785	PRJNA467785	SRP155515	SAMN09201524	Gp0266684	Illumina HiSeq-2000	2 × 151	12,009,368,844	MEGAHIT v1.1.2
81D	ocean_biome	ocean	water	Monterey Bay, CA, USA	10/11/2016	36.835 N, 121.901 W	100	Metagenome	1174656	1174756	81D metaG	166812	https://genome.jgi.doe.gov/portal/81DmetaG_FD/81DmetaG_FD.download.html	467762	PRJNA467762	SRP155407	SAMN09202075	Gp0266657	Illumina HiSeq-2000	2 × 151	8,013,377,022	SPAdes assembler 3.11.1
81R	ocean_biome	ocean	water	Monterey Bay, CA, USA	10/11/2016	36.835 N, 121.901 W	100	Metatranscriptome	1175060	1175112	81R metaT	166904	https://genome.jgi.doe.gov/portal/81RmetaT_FD/81RmetaT_FD.download.html	467786	PRJNA467786	SRP155517	SAMN09201523	Gp0266685	Illumina HiSeq-2000	2 × 151	10,557,788,932	MEGAHIT v1.1.2
82D	ocean_biome	ocean	water	Monterey Bay, CA, USA	10/10/2016	36.835 N, 121.901 W	100	Metagenome	1174658	1174757	82D metaG	166813	https://genome.jgi.doe.gov/portal/82DmetaG_FD/82DmetaG_FD.download.html	467763	PRJNA467763	SRP155413	SAMN09201536	Gp0266658	Illumina HiSeq-2000	2 × 151	5,810,730,358	SPAdes assembler 3.11.1
82R	ocean_biome	ocean	water	Monterey Bay, CA, USA	10/10/2016	36.835 N, 121.901 W	100	Metatranscriptome	1175063	1175113	82R metaT	166905	https://genome.jgi.doe.gov/portal/82RmetaT_FD/82RmetaT_FD.download.html	468150	PRJNA468150	SRP155535	SAMN09201528	Gp0267196	Illumina HiSeq-2000	2 × 151	16,445,750,354	MEGAHIT v1.1.2
84D	ocean_biome	ocean	water	Monterey Bay, CA, USA	10/11/2016	36.835 N, 121.901 W	100	Metagenome	1174660	1174758	84D metaG	166814	https://genome.jgi.doe.gov/portal/84DmetaG_FD/84DmetaG_FD.download.html	467764	PRJNA467764	SRP155419	SAMN09201535	Gp0266659	Illumina HiSeq-2000	2 × 151	7,118,809,232	SPAdes assembler 3.11.1
84R	ocean_biome	ocean	water	Monterey Bay, CA, USA	10/11/2016	36.835 N, 121.901 W	100	Metatranscriptome	1175066	1175114	84R metaT	166906	https://genome.jgi.doe.gov/portal/84RmetaT_FD/84RmetaT_FD.download.html	467787	PRJNA467787	SRP155539	SAMN09202120	Gp0266686	Illumina HiSeq-2000	2 × 151	12,527,235,424	MEGAHIT v1.1.2
85D	ocean_biome	ocean	water	Monterey Bay, CA, USA	10/13/2016	36.835 N, 121.901 W	100	Metagenome	1174662	1174759	85D metaG	166815	https://genome.jgi.doe.gov/portal/85DmetaG_FD/85DmetaG_FD.download.html	467765	PRJNA467765	SRP155420	SAMN09201686	Gp0266660	Illumina HiSeq-2000	2 × 151	7,178,048,646	SPAdes assembler 3.11.1
85R	ocean_biome	ocean	water	Monterey Bay, CA, USA	10/13/2016	36.835 N, 121.901 W	100	Metatranscriptome	1174912	1174972	85R metaT	166872	https://genome.jgi.doe.gov/portal/85RmetaT_FD/85RmetaT_FD.download.html	468329	PRJNA468329	SRP163292	SAMN09199668	Gp0290822	Illumina HiSeq-2000	2 × 151	8,012,925,230	MEGAHIT v1.1.2
89D	ocean_biome	ocean	water	Monterey Bay, CA, USA	11/5/2016	36.835 N, 121.901 W	575	Metagenome	1174664	1174760	89D metaG	166816	https://genome.jgi.doe.gov/portal/89DmetaG_FD/89DmetaG_FD.download.html	467766	PRJNA467766	SRP155421	SAMN09201534	Gp0266661	Illumina HiSeq-2000	2 × 151	7,163,560,498	SPAdes assembler 3.11.1
89R	ocean_biome	ocean	water	Monterey Bay, CA, USA	11/5/2016	36.835 N, 121.901 W	575	Metatranscriptome	1175069	1175115	89R metaT	166907	https://genome.jgi.doe.gov/portal/89RmetaT_FD/89RmetaT_FD.download.html	468151	PRJNA468151	SRP155534	SAMN09201527	Gp0267197	Illumina HiSeq-2000	2 × 151	12,255,826,816	MEGAHIT v1.1.2
8D	ocean_biome	ocean	water	Monterey Bay, CA, USA	11/5/2016	36.835 N, 121.901 W	775	Metagenome	1174516	1174686	8D metaG	166742	https://genome.jgi.doe.gov/portal/8DmetaG_FD/8DmetaG_FD.download.html	467724	PRJNA467724	SRP155246	SAMN09201853	Gp0266597	Illumina HiSeq-2000	2 × 151	9,439,829,628	SPAdes assembler 3.11.1
8R	ocean_biome	ocean	water	Monterey Bay, CA, USA	11/5/2016	36.835 N, 121.901 W	775	Metatranscriptome	1174780	1174928	8R metaT	166828	https://genome.jgi.doe.gov/portal/8RmetaT_FD/8RmetaT_FD.download.html	502451	PRJNA502451	SRP175188	SAMN10351181	Gp0290778	Illumina HiSeq-2000	2 × 151	7,929,114,492	MEGAHIT v1.1.2
90D	ocean_biome	ocean	water	Monterey Bay, CA, USA	11/8/2016	36.835 N, 121.901 W	175	Metagenome	1174666	1174761	90D metaG	166817	https://genome.jgi.doe.gov/portal/90DmetaG_FD/90DmetaG_FD.download.html	467768	PRJNA467768	SRP155424	SAMN09201440	Gp0266662	Illumina HiSeq-2000	2 × 151	10,726,324,864	SPAdes assembler 3.11.1
90R	ocean_biome	ocean	water	Monterey Bay, CA, USA	11/8/2016	36.835 N, 121.901 W	175	Metatranscriptome	1175072	1175116	90R metaT	166908	https://genome.jgi.doe.gov/portal/90RmetaT_FD/90RmetaT_FD.download.html	467788	PRJNA467788	SRP155602	SAMN09201435	Gp0266687	Illumina HiSeq-2000	2 × 151	11,924,015,188	MEGAHIT v1.1.2
91D	ocean_biome	ocean	water	Monterey Bay, CA, USA	11/11/2016	36.835 N, 121.901 W	475	Metagenome	1174668	1174762	91D metaG	166818	https://genome.jgi.doe.gov/portal/91DmetaG_FD/91DmetaG_FD.download.html	467769	PRJNA467769	SRP155425	SAMN09202113	Gp0266663	Illumina HiSeq-2000	2 × 151	6,496,286,666	SPAdes assembler 3.11.1
91R	ocean_biome	ocean	water	Monterey Bay, CA, USA	11/11/2016	36.835 N, 121.901 W	475	Metatranscriptome	1174915	1174973	91R metaT	166873	https://genome.jgi.doe.gov/portal/91RmetaT_FD/91RmetaT_FD.download.html	468330	PRJNA468330	SRP163297	SAMN09199667	Gp0290823	Illumina HiSeq-2000	2 × 151	9,122,005,734	MEGAHIT v1.1.2
9D	ocean_biome	ocean	water	Monterey Bay, CA, USA	11/15/2016	36.835 N, 121.901 W	975	Metagenome	1174518	1174687	9D metaG	166743	https://genome.jgi.doe.gov/portal/9DmetaG_FD/9DmetaG_FD.download.html	502408	PRJNA502408	SRP174088	SAMN10351267	Gp0266598	Illumina HiSeq-2000	2 × 151	10,053,837,002	SPAdes assembler 3.11.1
9R	ocean_biome	ocean	water	Monterey Bay, CA, USA	11/15/2016	36.835 N, 121.901 W	975	Metatranscriptome	1174783	1174929	9R metaT	166829	https://genome.jgi.doe.gov/portal/9RmetaT_FD/9RmetaT_FD.download.html	502452	PRJNA502452	SRP174123	SAMN10350901	Gp0290779	Illumina HiSeq-2000	2 × 151	11,212,078,576	MEGAHIT v1.1.2
MB_1025D_MT	ocean_biome	ocean	water	Monterey Bay, CA, USA	10/25/2016	36.835 N, 121.901 W	200	Metatranscriptome	1188669	1188681	MB_1025D metaT	175198	https://genome.jgi.doe.gov/portal/MB_1025DmetaT_FD/MB_1025DmetaT_FD.download.html	502611	PRJNA502611	SRP174142	SAMN10350584	Gp0294703	Illumina HiSeq-2000	2 × 151	30,614,719,084	MEGAHIT v1.1.2
MB_1026D_MT	ocean_biome	ocean	water	Monterey Bay, CA, USA	10/26/2016	36.835 N, 121.901 W	200	Metatranscriptome	1188672	1188682	MB_1026D metaT	175199	https://genome.jgi.doe.gov/portal/MB_1026DmetaT_FD/MB_1026DmetaT_FD.download.html	502612	PRJNA502612	SRP174143	SAMN10350798	Gp0294704	Illumina HiSeq-2000	2 × 151	37,885,516,762	MEGAHIT v1.1.2
WCR_12_MT	ocean_biome	ocean	water	Monterey Bay, CA, USA	9/26/2016	36.835 N, 121.901 W	200	Metatranscriptome	1188654	1188676	WCR_12 metaT	175193	https://genome.jgi.doe.gov/portal/WCR_12metaT_FD/WCR_12metaT_FD.download.html	502608	PRJNA502608	SRP174139	SAMN10350732	Gp0294698	Illumina HiSeq-2000	2 × 151	34,281,550,234	MEGAHIT v1.1.2
WCR_74_MT	ocean_biome	ocean	water	Monterey Bay, CA, USA	11/6/2016	36.835 N, 121.901 W	425	Metatranscriptome	1188657	1188677	WCR_74 metaT	175194	https://genome.jgi.doe.gov/portal/WCR_74metaT_FD/WCR_74metaT_FD.download.html	502609	PRJNA502609	SRP174140	SAMN10350604	Gp0294699	Illumina HiSeq-2000	2 × 151	36,435,144,246	MEGAHIT v1.1.2
WCR_77_MT	ocean_biome	ocean	water	Monterey Bay, CA, USA	11/7/2016	36.835 N, 121.901 W	625	Metatranscriptome	1188660	1188678	WCR_77 metaT	175195	https://genome.jgi.doe.gov/portal/WCR_77metaT_FD/WCR_77metaT_FD.download.html	502610	PRJNA502610	SRP174141	SAMN10350815	Gp0294700	Illumina HiSeq-2000	2 × 151	37,188,680,754	MEGAHIT v1.1.2
1_DF	ocean_biome	ocean	water	Monterey Bay, CA, USA	10/4/16	36.835 N, 121.901 W	200	Ribosomal iTag	1190879	1190881	Phytoplankton community itags pl1	178203		511260	PRJNA511260	SRP174784	SAMN10636594	Gp0358229	Illumina MiSeq	2 × 301	92,259,510	n/a
1_S	ocean_biome	ocean	water	Monterey Bay, CA, USA	10/4/16	36.835 N, 121.901 W	200	Ribosomal iTag	1190880	1190883	Bacterial community itags	178291		511216	PRJNA511216	SRP174685	SAMN10636692	Gp0358321	Illumina MiSeq	2 × 301	76,656,272	n/a
10_DF	ocean_biome	ocean	water	Monterey Bay, CA, USA	11/10/16	36.835 N, 121.901 W	350	Ribosomal iTag	1190879	1190881	Phytoplankton community itags pl1	178211		511268	PRJNA511268	SRP174801	SAMN10636691	Gp0358237	Illumina MiSeq	2 × 301	104,195,966	n/a
10_S	ocean_biome	ocean	water	Monterey Bay, CA, USA	11/10/16	36.835 N, 121.901 W	350	Ribosomal iTag	1190880	1190883	Bacterial community itags	178299		511224	PRJNA511224	SRP174679	SAMN10636635	Gp0358330	Illumina MiSeq	2 × 301	105,784,042	n/a
11_DF	ocean_biome	ocean	water	Monterey Bay, CA, USA	10/6/16	36.835 N, 121.901 W	250	Ribosomal iTag	1190879	1190881	Phytoplankton community itags pl1	178212		511269	PRJNA511269	SRP174800	SAMN10636604	Gp0358238	Illumina MiSeq	2 × 301	86,498,370	n/a
11_S	ocean_biome	ocean	water	Monterey Bay, CA, USA	10/6/16	36.835 N, 121.901 W	250	Ribosomal iTag	1190880	1190883	Bacterial community itags	178300		511225	PRJNA511225	SRP174672	SAMN10636702	Gp0358332	Illumina MiSeq	2 × 301	7,791,686	n/a
12_DF	ocean_biome	ocean	water	Monterey Bay, CA, USA	9/29/16	36.835 N, 121.901 W	125	Ribosomal iTag	1190879	1190881	Phytoplankton community itags pl1	178213		511270	PRJNA511270	SRP174803	SAMN10636591	Gp0358239	Illumina MiSeq	2 × 301	92,700,174	n/a
12_S	ocean_biome	ocean	water	Monterey Bay, CA, USA	9/29/16	36.835 N, 121.901 W	125	Ribosomal iTag	1190880	1190883	Bacterial community itags	178301		511226	PRJNA511226	SRP174673	SAMN10636675	Gp0358333	Illumina MiSeq	2 × 301	56,683,718	n/a
125r_DF	ocean_biome	ocean	water	Monterey Bay, CA, USA	11/15/16	36.835 N, 121.901 W	950	Ribosomal iTag	1190879	1190881	Phytoplankton community itags pl1	178290		511215	PRJNA511215	SRP174796	SAMN10636674	Gp0358320	Illumina MiSeq	2 × 301	106,222,298	n/a
125r_S	ocean_biome	ocean	water	Monterey Bay, CA, USA	11/15/16	36.835 N, 121.901 W	950	Ribosomal iTag	1190880	1190883	Bacterial community itags	178378		511162	PRJNA511162	SRP174734	SAMN10637061	Gp0358415	Illumina MiSeq	2 × 301	962,598	n/a
13_DF	ocean_biome	ocean	water	Monterey Bay, CA, USA	10/1/16	36.835 N, 121.901 W	125	Ribosomal iTag	1190879	1190881	Phytoplankton community itags pl1	178214		511271	PRJNA511271	SRP174843	SAMN10636660	Gp0358240	Illumina MiSeq	2 × 301	102,382,140	n/a
13_S	ocean_biome	ocean	water	Monterey Bay, CA, USA	10/1/16	36.835 N, 121.901 W	125	Ribosomal iTag	1190880	1190883	Bacterial community itags	178302		511227	PRJNA511227	SRP174671	SAMN10636634	Gp0358334	Illumina MiSeq	2 × 301	51,512,538	n/a
14_DF	ocean_biome	ocean	water	Monterey Bay, CA, USA	10/28/16	36.835 N, 121.901 W	250	Ribosomal iTag	1190879	1190881	Phytoplankton community itags pl1	178215		511272	PRJNA511272	SRP174793	SAMN10636590	Gp0358241	Illumina MiSeq	2 × 301	105,082,110	n/a
14_S	ocean_biome	ocean	water	Monterey Bay, CA, USA	10/28/16	36.835 N, 121.901 W	250	Ribosomal iTag	1190880	1190883	Bacterial community itags	178303		511228	PRJNA511228	SRP174676	SAMN10636701	Gp0358335	Illumina MiSeq	2 × 301	87,379,698	n/a
15_DF	ocean_biome	ocean	water	Monterey Bay, CA, USA	10/13/16	36.835 N, 121.901 W	100	Ribosomal iTag	1190879	1190881	Phytoplankton community itags pl1	178216		511273	PRJNA511273	SRP174791	SAMN10636605	Gp0358243	Illumina MiSeq	2 × 301	101,095,666	n/a
15_S	ocean_biome	ocean	water	Monterey Bay, CA, USA	10/13/16	36.835 N, 121.901 W	100	Ribosomal iTag	1190880	1190883	Bacterial community itags	178304		511229	PRJNA511229	SRP174674	SAMN10636700	Gp0358336	Illumina MiSeq	2 × 301	76,358,282	n/a
16_DF	ocean_biome	ocean	water	Monterey Bay, CA, USA	10/17/16	36.835 N, 121.901 W	200	Ribosomal iTag	1190879	1190881	Phytoplankton community itags pl1	178217		511274	PRJNA511274	SRP174790	SAMN10636960	Gp0358244	Illumina MiSeq	2 × 301	125,671,714	n/a
16_S	ocean_biome	ocean	water	Monterey Bay, CA, USA	10/17/16	36.835 N, 121.901 W	200	Ribosomal iTag	1190880	1190883	Bacterial community itags	178305		511230	PRJNA511230	SRP174675	SAMN10636714	Gp0358337	Illumina MiSeq	2 × 301	81,619,762	n/a
17_DF	ocean_biome	ocean	water	Monterey Bay, CA, USA	11/12/16	36.835 N, 121.901 W	400	Ribosomal iTag	1190879	1190881	Phytoplankton community itags pl1	178218		511275	PRJNA511275	SRP174792	SAMN10636958	Gp0358245	Illumina MiSeq	2 × 301	98,466,732	n/a
17_S	ocean_biome	ocean	water	Monterey Bay, CA, USA	11/12/16	36.835 N, 121.901 W	400	Ribosomal iTag	1190880	1190883	Bacterial community itags	178306		511231	PRJNA511231	SRP174677	SAMN10636699	Gp0358338	Illumina MiSeq	2 × 301	87,798,088	n/a
18_DF	ocean_biome	ocean	water	Monterey Bay, CA, USA	10/20/16	36.835 N, 121.901 W	200	Ribosomal iTag	1190879	1190881	Phytoplankton community itags pl1	178219		511276	PRJNA511276	SRP174795	SAMN10636606	Gp0358246	Illumina MiSeq	2 × 301	119,638,470	n/a
18_S	ocean_biome	ocean	water	Monterey Bay, CA, USA	10/20/16	36.835 N, 121.901 W	200	Ribosomal iTag	1190880	1190883	Bacterial community itags	178307		511232	PRJNA511232	SRP174701	SAMN10636698	Gp0358339	Illumina MiSeq	2 × 301	81,561,970	n/a
1r_DF	ocean_biome	ocean	water	Monterey Bay, CA, USA	9/26/16	36.835 N, 121.901 W	95.1	Ribosomal iTag	1190879	1190881	Phytoplankton community itags pl1	178286		511211	PRJNA511211	SRP174819	SAMN10636639	Gp0358316	Illumina MiSeq	2 × 301	92,464,190	n/a
1r_S	ocean_biome	ocean	water	Monterey Bay, CA, USA	9/26/16	36.835 N, 121.901 W	95.1	Ribosomal iTag	1190880	1190883	Bacterial community itags	178374		511158	PRJNA511158	SRP174753	SAMN10636615	Gp0358410	Illumina MiSeq	2 × 301	23,979,466	n/a
2_DF	ocean_biome	ocean	water	Monterey Bay, CA, USA	11/16/16	36.835 N, 121.901 W	1000	Ribosomal iTag	1190879	1190881	Phytoplankton community itags pl1	178204		511261	PRJNA511261	SRP174785	SAMN10636593	Gp0358230	Illumina MiSeq	2 × 301	100,366,644	n/a
2_S	ocean_biome	ocean	water	Monterey Bay, CA, USA	11/16/16	36.835 N, 121.901 W	1000	Ribosomal iTag	1190880	1190883	Bacterial community itags	178292		511217	PRJNA511217	SRP174686	SAMN10636930	Gp0358322	Illumina MiSeq	2 × 301	70,867,440	n/a
20_DF	ocean_biome	ocean	water	Monterey Bay, CA, USA	9/27/16	36.835 N, 121.901 W	73.5	Ribosomal iTag	1190879	1190881	Phytoplankton community itags pl1	178220		511277	PRJNA511277	SRP174794	SAMN10636665	Gp0358247	Illumina MiSeq	2 × 301	76,705,636	n/a
20_S	ocean_biome	ocean	water	Monterey Bay, CA, USA	9/27/16	36.835 N, 121.901 W	73.5	Ribosomal iTag	1190880	1190883	Bacterial community itags	178308		511233	PRJNA511233	SRP174733	SAMN10636697	Gp0358340	Illumina MiSeq	2 × 301	2,140,712	n/a
21_DF	ocean_biome	ocean	water	Monterey Bay, CA, USA	10/24/16	36.835 N, 121.901 W	250	Ribosomal iTag	1190879	1190881	Phytoplankton community itags pl1	178221		511278	PRJNA511278	SRP174797	SAMN10636659	Gp0358248	Illumina MiSeq	2 × 301	132,824,076	n/a
21_S	ocean_biome	ocean	water	Monterey Bay, CA, USA	10/24/16	36.835 N, 121.901 W	250	Ribosomal iTag	1190880	1190883	Bacterial community itags	178309		511234	PRJNA511234	SRP174732	SAMN10636715	Gp0358341	Illumina MiSeq	2 × 301	102,678,324	n/a
22_DF	ocean_biome	ocean	water	Monterey Bay, CA, USA	10/12/16	36.835 N, 121.901 W	100	Ribosomal iTag	1190879	1190881	Phytoplankton community itags pl1	178222		511279	PRJNA511279	SRP174821	SAMN10636709	Gp0358249	Illumina MiSeq	2 × 301	87,594,010	n/a
22_S	ocean_biome	ocean	water	Monterey Bay, CA, USA	10/12/16	36.835 N, 121.901 W	100	Ribosomal iTag	1190880	1190883	Bacterial community itags	178310		511235	PRJNA511235	SRP174738	SAMN10636609	Gp0358342	Illumina MiSeq	2 × 301	75,881,498	n/a
23_DF	ocean_biome	ocean	water	Monterey Bay, CA, USA	10/6/16	36.835 N, 121.901 W	150	Ribosomal iTag	1190879	1190881	Phytoplankton community itags pl1	178223		511280	PRJNA511280	SRP174820	SAMN10636658	Gp0358250	Illumina MiSeq	2 × 301	119,028,644	n/a
23_S	ocean_biome	ocean	water	Monterey Bay, CA, USA	10/6/16	36.835 N, 121.901 W	150	Ribosomal iTag	1190880	1190883	Bacterial community itags	178311		511236	PRJNA511236	SRP174740	SAMN10636633	Gp0358343	Illumina MiSeq	2 × 301	72,300,200	n/a
24_DF	ocean_biome	ocean	water	Monterey Bay, CA, USA	11/2/16	36.835 N, 121.901 W	425	Ribosomal iTag	1190879	1190881	Phytoplankton community itags pl1	178224		511281	PRJNA511281	SRP174823	SAMN10636666	Gp0358251	Illumina MiSeq	2 × 301	106,448,650	n/a
24_S	ocean_biome	ocean	water	Monterey Bay, CA, USA	11/2/16	36.835 N, 121.901 W	425	Ribosomal iTag	1190880	1190883	Bacterial community itags	178312		511237	PRJNA511237	SRP174735	SAMN10636922	Gp0358345	Illumina MiSeq	2 × 301	61,332,362	n/a
25_DF	ocean_biome	ocean	water	Monterey Bay, CA, USA	11/6/16	36.835 N, 121.901 W	575	Ribosomal iTag	1190879	1190881	Phytoplankton community itags pl1	178225		511282	PRJNA511282	SRP174824	SAMN10636657	Gp0358252	Illumina MiSeq	2 × 301	117,012,546	n/a
25_S	ocean_biome	ocean	water	Monterey Bay, CA, USA	11/6/16	36.835 N, 121.901 W	575	Ribosomal iTag	1190880	1190883	Bacterial community itags	178313		511238	PRJNA511238	SRP174700	SAMN10636920	Gp0358346	Illumina MiSeq	2 × 301	103,392,898	n/a
26_DF	ocean_biome	ocean	water	Monterey Bay, CA, USA	10/1/16	36.835 N, 121.901 W	125	Ribosomal iTag	1190879	1190881	Phytoplankton community itags pl1	178226		511283	PRJNA511283	SRP174822	SAMN10636667	Gp0358253	Illumina MiSeq	2 × 301	129,192,210	n/a
26_S	ocean_biome	ocean	water	Monterey Bay, CA, USA	10/1/16	36.835 N, 121.901 W	125	Ribosomal iTag	1190880	1190883	Bacterial community itags	178314		511239	PRJNA511239	SRP174702	SAMN10636610	Gp0358347	Illumina MiSeq	2 × 301	80,336,900	n/a
27_DF	ocean_biome	ocean	water	Monterey Bay, CA, USA	10/12/16	36.835 N, 121.901 W	100	Ribosomal iTag	1190879	1190881	Phytoplankton community itags pl1	178227		511284	PRJNA511284	SRP174826	SAMN10636957	Gp0358254	Illumina MiSeq	2 × 301	98,704,522	n/a
27_S	ocean_biome	ocean	water	Monterey Bay, CA, USA	10/12/16	36.835 N, 121.901 W	100	Ribosomal iTag	1190880	1190883	Bacterial community itags	178315		511240	PRJNA511240	SRP174737	SAMN10636696	Gp0358348	Illumina MiSeq	2 × 301	101,889,704	n/a
28_DF	ocean_biome	ocean	water	Monterey Bay, CA, USA	10/4/16	36.835 N, 121.901 W	149.5	Ribosomal iTag	1190879	1190881	Phytoplankton community itags pl1	178228		511285	PRJNA511285	SRP174827	SAMN10636595	Gp0358255	Illumina MiSeq	2 × 301	116,352,152	n/a
28_S	ocean_biome	ocean	water	Monterey Bay, CA, USA	10/4/16	36.835 N, 121.901 W	149.5	Ribosomal iTag	1190880	1190883	Bacterial community itags	178316		511241	PRJNA511241	SRP174736	SAMN10636689	Gp0358349	Illumina MiSeq	2 × 301	74,780,440	n/a
29_DF	ocean_biome	ocean	water	Monterey Bay, CA, USA	10/10/16	36.835 N, 121.901 W	100	Ribosomal iTag	1190879	1190881	Phytoplankton community itags pl1	178229		511286	PRJNA511286	SRP174825	SAMN10636956	Gp0358256	Illumina MiSeq	2 × 301	100,751,322	n/a
29_S	ocean_biome	ocean	water	Monterey Bay, CA, USA	10/10/16	36.835 N, 121.901 W	100	Ribosomal iTag	1190880	1190883	Bacterial community itags	178317		511163	PRJNA511163	SRP174699	SAMN10636676	Gp0358350	Illumina MiSeq	2 × 301	85,079,456	n/a
3_DF	ocean_biome	ocean	water	Monterey Bay, CA, USA	11/9/16	36.835 N, 121.901 W	175	Ribosomal iTag	1190879	1190881	Phytoplankton community itags pl1	178205		511262	PRJNA511262	SRP174781	SAMN10636603	Gp0358231	Illumina MiSeq	2 × 301	57,795,612	n/a
3_S	ocean_biome	ocean	water	Monterey Bay, CA, USA	11/9/16	36.835 N, 121.901 W	175	Ribosomal iTag	1190880	1190883	Bacterial community itags	178293		511218	PRJNA511218	SRP174680	SAMN10637051	Gp0358323	Illumina MiSeq	2 × 301	66,809,358	n/a
30_DF	ocean_biome	ocean	water	Monterey Bay, CA, USA	10/5/16	36.835 N, 121.901 W	200	Ribosomal iTag	1190879	1190881	Phytoplankton community itags pl1	178230		511287	PRJNA511287	SRP174817	SAMN10636954	Gp0358257	Illumina MiSeq	2 × 301	108,967,418	n/a
30_S	ocean_biome	ocean	water	Monterey Bay, CA, USA	10/5/16	36.835 N, 121.901 W	200	Ribosomal iTag	1190880	1190883	Bacterial community itags	178318		511164	PRJNA511164	SRP174720	SAMN10636632	Gp0358351	Illumina MiSeq	2 × 301	85,140,258	n/a
31_DF	ocean_biome	ocean	water	Monterey Bay, CA, USA	10/26/16	36.835 N, 121.901 W	200	Ribosomal iTag	1190879	1190881	Phytoplankton community itags pl1	178231		511288	PRJNA511288	SRP174847	SAMN10636596	Gp0358258	Illumina MiSeq	2 × 301	98,156,100	n/a
31_S	ocean_biome	ocean	water	Monterey Bay, CA, USA	10/26/16	36.835 N, 121.901 W	200	Ribosomal iTag	1190880	1190883	Bacterial community itags	178319		511165	PRJNA511165	SRP174722	SAMN10636631	Gp0358352	Illumina MiSeq	2 × 301	65,760,674	n/a
32_DF	ocean_biome	ocean	water	Monterey Bay, CA, USA	10/19/16	36.835 N, 121.901 W	200	Ribosomal iTag	1190879	1190881	Phytoplankton community itags pl1	178232		511289	PRJNA511289	SRP174845	SAMN10636656	Gp0358259	Illumina MiSeq	2 × 301	108,968,622	n/a
32_S	ocean_biome	ocean	water	Monterey Bay, CA, USA	10/19/16	36.835 N, 121.901 W	200	Ribosomal iTag	1190880	1190883	Bacterial community itags	178320		511166	PRJNA511166	SRP174714	SAMN10636918	Gp0358353	Illumina MiSeq	2 × 301	54,899,992	n/a
33_DF	ocean_biome	ocean	water	Monterey Bay, CA, USA	9/28/16	36.835 N, 121.901 W	75	Ribosomal iTag	1190879	1190881	Phytoplankton community itags pl1	178233		511290	PRJNA511290	SRP174816	SAMN10636655	Gp0358260	Illumina MiSeq	2 × 301	130,152,400	n/a
33_S	ocean_biome	ocean	water	Monterey Bay, CA, USA	9/28/16	36.835 N, 121.901 W	75	Ribosomal iTag	1190880	1190883	Bacterial community itags	178321		511167	PRJNA511167	SRP174684	SAMN10636916	Gp0358354	Illumina MiSeq	2 × 301	110,829,404	n/a
34_DF	ocean_biome	ocean	water	Monterey Bay, CA, USA	9/30/16	36.835 N, 121.901 W	94	Ribosomal iTag	1190879	1190881	Phytoplankton community itags pl1	178234		511291	PRJNA511291	SRP174841	SAMN10636668	Gp0358261	Illumina MiSeq	2 × 301	150,864,812	n/a
34_S	ocean_biome	ocean	water	Monterey Bay, CA, USA	9/30/16	36.835 N, 121.901 W	94	Ribosomal iTag	1190880	1190883	Bacterial community itags	178322		511168	PRJNA511168	SRP174713	SAMN10637055	Gp0358355	Illumina MiSeq	2 × 301	95,840,206	n/a
35_DF	ocean_biome	ocean	water	Monterey Bay, CA, USA	10/20/16	36.835 N, 121.901 W	200	Ribosomal iTag	1190879	1190881	Phytoplankton community itags pl1	178235		511292	PRJNA511292	SRP174839	SAMN10636654	Gp0358262	Illumina MiSeq	2 × 301	111,955,746	n/a
35_S	ocean_biome	ocean	water	Monterey Bay, CA, USA	10/20/16	36.835 N, 121.901 W	200	Ribosomal iTag	1190880	1190883	Bacterial community itags	178323		511169	PRJNA511169	SRP174715	SAMN10636630	Gp0358356	Illumina MiSeq	2 × 301	88,798,612	n/a
36_DF	ocean_biome	ocean	water	Monterey Bay, CA, USA	11/14/16	36.835 N, 121.901 W	825	Ribosomal iTag	1190879	1190881	Phytoplankton community itags pl1	178236		511293	PRJNA511293	SRP174838	SAMN10636669	Gp0358264	Illumina MiSeq	2 × 301	102,925,746	n/a
36_S	ocean_biome	ocean	water	Monterey Bay, CA, USA	11/14/16	36.835 N, 121.901 W	825	Ribosomal iTag	1190880	1190883	Bacterial community itags	178324		511170	PRJNA511170	SRP174712	SAMN10636677	Gp0358358	Illumina MiSeq	2 × 301	82,905,032	n/a
36r_DF	ocean_biome	ocean	water	Monterey Bay, CA, USA	10/5/16	36.835 N, 121.901 W	200	Ribosomal iTag	1190879	1190881	Phytoplankton community itags pl1	178287		511212	PRJNA511212	SRP174787	SAMN10636638	Gp0358317	Illumina MiSeq	2 × 301	146,827,800	n/a
36r_S	ocean_biome	ocean	water	Monterey Bay, CA, USA	10/5/16	36.835 N, 121.901 W	200	Ribosomal iTag	1190880	1190883	Bacterial community itags	178375		511159	PRJNA511159	SRP174747	SAMN10636685	Gp0358412	Illumina MiSeq	2 × 301	29,935,654	n/a
37_DF	ocean_biome	ocean	water	Monterey Bay, CA, USA	10/25/16	36.835 N, 121.901 W	200	Ribosomal iTag	1190879	1190881	Phytoplankton community itags pl1	178237		511294	PRJNA511294	SRP174811	SAMN10636708	Gp0358265	Illumina MiSeq	2 × 301	116,812,682	n/a
37_S	ocean_biome	ocean	water	Monterey Bay, CA, USA	10/25/16	36.835 N, 121.901 W	200	Ribosomal iTag	1190880	1190883	Bacterial community itags	178325		511171	PRJNA511171	SRP174717	SAMN10636914	Gp0358359	Illumina MiSeq	2 × 301	93,110,136	n/a
38_DF	ocean_biome	ocean	water	Monterey Bay, CA, USA	10/2/16	36.835 N, 121.901 W	175	Ribosomal iTag	1190879	1190881	Phytoplankton community itags pl1	178238		511295	PRJNA511295	SRP174810	SAMN10636953	Gp0358266	Illumina MiSeq	2 × 301	115,647,812	n/a
38_S	ocean_biome	ocean	water	Monterey Bay, CA, USA	10/2/16	36.835 N, 121.901 W	175	Ribosomal iTag	1190880	1190883	Bacterial community itags	178326		511172	PRJNA511172	SRP174730	SAMN10636716	Gp0358360	Illumina MiSeq	2 × 301	61,039,188	n/a
39_DF	ocean_biome	ocean	water	Monterey Bay, CA, USA	9/28/16	36.835 N, 121.901 W	75	Ribosomal iTag	1190879	1190881	Phytoplankton community itags pl1	178239		511296	PRJNA511296	SRP174818	SAMN10636597	Gp0358267	Illumina MiSeq	2 × 301	140,738,570	n/a
39_S	ocean_biome	ocean	water	Monterey Bay, CA, USA	9/28/16	36.835 N, 121.901 W	75	Ribosomal iTag	1190880	1190883	Bacterial community itags	178327		511173	PRJNA511173	SRP174719	SAMN10636695	Gp0358361	Illumina MiSeq	2 × 301	46,600,218	n/a
4_DF	ocean_biome	ocean	water	Monterey Bay, CA, USA	11/3/16	36.835 N, 121.901 W	325	Ribosomal iTag	1190879	1190881	Phytoplankton community itags pl1	178206		511263	PRJNA511263	SRP174799	SAMN10636592	Gp0358232	Illumina MiSeq	2 × 301	84,373,912	n/a
4_S	ocean_biome	ocean	water	Monterey Bay, CA, USA	11/3/16	36.835 N, 121.901 W	325	Ribosomal iTag	1190880	1190883	Bacterial community itags	178294		511219	PRJNA511219	SRP174687	SAMN10636928	Gp0358325	Illumina MiSeq	2 × 301	61,727,274	n/a
40_DF	ocean_biome	ocean	water	Monterey Bay, CA, USA	11/4/16	36.835 N, 121.901 W	400	Ribosomal iTag	1190879	1190881	Phytoplankton community itags pl1	178240		511297	PRJNA511297	SRP174808	SAMN10636710	Gp0358268	Illumina MiSeq	2 × 301	120,755,782	n/a
40_S	ocean_biome	ocean	water	Monterey Bay, CA, USA	11/4/16	36.835 N, 121.901 W	400	Ribosomal iTag	1190880	1190883	Bacterial community itags	178328		511174	PRJNA511174	SRP174718	SAMN10636694	Gp0358362	Illumina MiSeq	2 × 301	69,728,456	n/a
41_DF	ocean_biome	ocean	water	Monterey Bay, CA, USA	11/13/16	36.835 N, 121.901 W	350	Ribosomal iTag	1190879	1190881	Phytoplankton community itags pl1	178241		511298	PRJNA511298	SRP174809	SAMN10636653	Gp0358269	Illumina MiSeq	2 × 301	153,685,784	n/a
41_S	ocean_biome	ocean	water	Monterey Bay, CA, USA	11/13/16	36.835 N, 121.901 W	350	Ribosomal iTag	1190880	1190883	Bacterial community itags	178329		511175	PRJNA511175	SRP174716	SAMN10636693	Gp0358363	Illumina MiSeq	2 × 301	105,190,470	n/a
42_DF	ocean_biome	ocean	water	Monterey Bay, CA, USA	9/27/16	36.835 N, 121.901 W	75	Ribosomal iTag	1190879	1190881	Phytoplankton community itags pl1	178242		511299	PRJNA511299	SRP174813	SAMN10636652	Gp0358270	Illumina MiSeq	2 × 301	120,916,516	n/a
42_S	ocean_biome	ocean	water	Monterey Bay, CA, USA	9/27/16	36.835 N, 121.901 W	75	Ribosomal iTag	1190880	1190883	Bacterial community itags	178330		511176	PRJNA511176	SRP174731	SAMN10636601	Gp0358364	Illumina MiSeq	2 × 301	66,539,060	n/a
43_DF	ocean_biome	ocean	water	Monterey Bay, CA, USA	11/8/16	36.835 N, 121.901 W	225	Ribosomal iTag	1190879	1190881	Phytoplankton community itags pl1	178243		511300	PRJNA511300	SRP174814	SAMN10636951	Gp0358271	Illumina MiSeq	2 × 301	161,805,560	n/a
43_S	ocean_biome	ocean	water	Monterey Bay, CA, USA	11/8/16	36.835 N, 121.901 W	225	Ribosomal iTag	1190880	1190883	Bacterial community itags	178331		511177	PRJNA511177	SRP174724	SAMN10636912	Gp0358365	Illumina MiSeq	2 × 301	64,745,702	n/a
44_DF	ocean_biome	ocean	water	Monterey Bay, CA, USA	9/30/16	36.835 N, 121.901 W	94.1	Ribosomal iTag	1190879	1190881	Phytoplankton community itags pl1	178244		511301	PRJNA511301	SRP174815	SAMN10636949	Gp0358272	Illumina MiSeq	2 × 301	107,242,688	n/a
44_S	ocean_biome	ocean	water	Monterey Bay, CA, USA	9/30/16	36.835 N, 121.901 W	94.1	Ribosomal iTag	1190880	1190883	Bacterial community itags	178332		511178	PRJNA511178	SRP174721	SAMN10636911	Gp0358366	Illumina MiSeq	2 × 301	81,440,366	n/a
45_DF	ocean_biome	ocean	water	Monterey Bay, CA, USA	11/3/16	36.835 N, 121.901 W	300	Ribosomal iTag	1190879	1190881	Phytoplankton community itags pl1	178245		511302	PRJNA511302	SRP174831	SAMN10636598	Gp0358273	Illumina MiSeq	2 × 301	90,206,690	n/a
45_S	ocean_biome	ocean	water	Monterey Bay, CA, USA	11/3/16	36.835 N, 121.901 W	300	Ribosomal iTag	1190880	1190883	Bacterial community itags	178333		511179	PRJNA511179	SRP174726	SAMN10637056	Gp0358367	Illumina MiSeq	2 × 301	100,148,720	n/a
46_DF	ocean_biome	ocean	water	Monterey Bay, CA, USA	10/21/16	36.835 N, 121.901 W	200	Ribosomal iTag	1190879	1190881	Phytoplankton community itags pl1	178246		511303	PRJNA511303	SRP174830	SAMN10636651	Gp0358274	Illumina MiSeq	2 × 301	60,635,848	n/a
46_S	ocean_biome	ocean	water	Monterey Bay, CA, USA	10/21/16	36.835 N, 121.901 W	200	Ribosomal iTag	1190880	1190883	Bacterial community itags	178334		511180	PRJNA511180	SRP174723	SAMN10636629	Gp0358368	Illumina MiSeq	2 × 301	87,755,346	n/a
47_DF	ocean_biome	ocean	water	Monterey Bay, CA, USA	10/25/16	36.835 N, 121.901 W	200	Ribosomal iTag	1190879	1190881	Phytoplankton community itags pl1	178247		511304	PRJNA511304	SRP174837	SAMN10636707	Gp0358275	Illumina MiSeq	2 × 301	115,867,542	n/a
47_S	ocean_biome	ocean	water	Monterey Bay, CA, USA	10/25/16	36.835 N, 121.901 W	200	Ribosomal iTag	1190880	1190883	Bacterial community itags	178335		511181	PRJNA511181	SRP174725	SAMN10636628	Gp0358369	Illumina MiSeq	2 × 301	66,638,390	n/a
48_DF	ocean_biome	ocean	water	Monterey Bay, CA, USA	10/31/16	36.835 N, 121.901 W	300	Ribosomal iTag	1190879	1190881	Phytoplankton community itags pl1	178248		511305	PRJNA511305	SRP174828	SAMN10636711	Gp0358276	Illumina MiSeq	2 × 301	65,209,242	n/a
48_S	ocean_biome	ocean	water	Monterey Bay, CA, USA	10/31/16	36.835 N, 121.901 W	300	Ribosomal iTag	1190880	1190883	Bacterial community itags	178336		511182	PRJNA511182	SRP174728	SAMN10636678	Gp0358371	Illumina MiSeq	2 × 301	74,671,478	n/a
48r_DF	ocean_biome	ocean	water	Monterey Bay, CA, USA	11/2/16	36.835 N, 121.901 W	550	Ribosomal iTag	1190879	1190881	Phytoplankton community itags pl1	178288		511213	PRJNA511213	SRP174782	SAMN10636673	Gp0358318	Illumina MiSeq	2 × 301	127,629,418	n/a
48r_S	ocean_biome	ocean	water	Monterey Bay, CA, USA	11/2/16	36.835 N, 121.901 W	550	Ribosomal iTag	1190880	1190883	Bacterial community itags	178376		511160	PRJNA511160	SRP174748	SAMN10636614	Gp0358413	Illumina MiSeq	2 × 301	58,653,462	n/a
49_DF	ocean_biome	ocean	water	Monterey Bay, CA, USA	10/3/16	36.835 N, 121.901 W	325	Ribosomal iTag	1190879	1190881	Phytoplankton community itags pl1	178249		511306	PRJNA511306	SRP174846	SAMN10636706	Gp0358277	Illumina MiSeq	2 × 301	95,586,764	n/a
49_S	ocean_biome	ocean	water	Monterey Bay, CA, USA	10/3/16	36.835 N, 121.901 W	325	Ribosomal iTag	1190880	1190883	Bacterial community itags	178337		511183	PRJNA511183	SRP174729	SAMN10636627	Gp0358372	Illumina MiSeq	2 × 301	83,797,798	n/a
5_DF	ocean_biome	ocean	water	Monterey Bay, CA, USA	10/19/16	36.835 N, 121.901 W	200	Ribosomal iTag	1190879	1190881	Phytoplankton community itags pl1	178207		511264	PRJNA511264	SRP174798	SAMN10636664	Gp0358233	Illumina MiSeq	2 × 301	44,540,776	n/a
5_S	ocean_biome	ocean	water	Monterey Bay, CA, USA	10/19/16	36.835 N, 121.901 W	200	Ribosomal iTag	1190880	1190883	Bacterial community itags	178295		511220	PRJNA511220	SRP174681	SAMN10636926	Gp0358326	Illumina MiSeq	2 × 301	30,100,602	n/a
50_DF	ocean_biome	ocean	water	Monterey Bay, CA, USA	10/18/16	36.835 N, 121.901 W	200	Ribosomal iTag	1190879	1190881	Phytoplankton community itags pl1	178250		511307	PRJNA511307	SRP174836	SAMN10636705	Gp0358278	Illumina MiSeq	2 × 301	84,935,578	n/a
50_S	ocean_biome	ocean	water	Monterey Bay, CA, USA	10/18/16	36.835 N, 121.901 W	200	Ribosomal iTag	1190880	1190883	Bacterial community itags	178338		511184	PRJNA511184	SRP174727	SAMN10636679	Gp0358373	Illumina MiSeq	2 × 301	93,846,382	n/a
51_DF	ocean_biome	ocean	water	Monterey Bay, CA, USA	11/13/16	36.835 N, 121.901 W	400	Ribosomal iTag	1190879	1190881	Phytoplankton community itags pl1	178251		511308	PRJNA511308	SRP174844	SAMN10636650	Gp0358279	Illumina MiSeq	2 × 301	108,360	n/a
51_S	ocean_biome	ocean	water	Monterey Bay, CA, USA	11/13/16	36.835 N, 121.901 W	400	Ribosomal iTag	1190880	1190883	Bacterial community itags	178339		511185	PRJNA511185	SRP174710	SAMN10636626	Gp0358374	Illumina MiSeq	2 × 301	103,485,606	n/a
52_DF	ocean_biome	ocean	water	Monterey Bay, CA, USA	10/17/16	36.835 N, 121.901 W	200	Ribosomal iTag	1190879	1190881	Phytoplankton community itags pl1	178252		511309	PRJNA511309	SRP174833	SAMN10636670	Gp0358280	Illumina MiSeq	2 × 301	98,717,766	n/a
52_S	ocean_biome	ocean	water	Monterey Bay, CA, USA	10/17/16	36.835 N, 121.901 W	200	Ribosomal iTag	1190880	1190883	Bacterial community itags	178340		511186	PRJNA511186	SRP174706	SAMN10636625	Gp0358375	Illumina MiSeq	2 × 301	84,900,662	n/a
53_DF	ocean_biome	ocean	water	Monterey Bay, CA, USA	9/29/16	36.835 N, 121.901 W	125	Ribosomal iTag	1190879	1190881	Phytoplankton community itags pl1	178253		511310	PRJNA511310	SRP174832	SAMN10636947	Gp0358281	Illumina MiSeq	2 × 301	110,774,622	n/a
53_S	ocean_biome	ocean	water	Monterey Bay, CA, USA	9/29/16	36.835 N, 121.901 W	125	Ribosomal iTag	1190880	1190883	Bacterial community itags	178341		511187	PRJNA511187	SRP174711	SAMN10636680	Gp0358376	Illumina MiSeq	2 × 301	86,629,606	n/a
54_DF	ocean_biome	ocean	water	Monterey Bay, CA, USA	11/11/16	36.835 N, 121.901 W	425	Ribosomal iTag	1190879	1190881	Phytoplankton community itags pl1	178254		511311	PRJNA511311	SRP174773	SAMN10636712	Gp0358282	Illumina MiSeq	2 × 301	62,542,984	n/a
54_S	ocean_biome	ocean	water	Monterey Bay, CA, USA	11/11/16	36.835 N, 121.901 W	425	Ribosomal iTag	1190880	1190883	Bacterial community itags	178342		511188	PRJNA511188	SRP174708	SAMN10636624	Gp0358377	Illumina MiSeq	2 × 301	80,089,478	n/a
55_DF	ocean_biome	ocean	water	Monterey Bay, CA, USA	11/8/16	36.835 N, 121.901 W	175	Ribosomal iTag	1190879	1190881	Phytoplankton community itags pl1	178255		511312	PRJNA511312	SRP174768	SAMN10636704	Gp0358284	Illumina MiSeq	2 × 301	62,783,182	n/a
55_S	ocean_biome	ocean	water	Monterey Bay, CA, USA	11/8/16	36.835 N, 121.901 W	175	Ribosomal iTag	1190880	1190883	Bacterial community itags	178343		511189	PRJNA511189	SRP174709	SAMN10636623	Gp0358378	Illumina MiSeq	2 × 301	48,110,636	n/a
56_DF	ocean_biome	ocean	water	Monterey Bay, CA, USA	11/9/16	36.835 N, 121.901 W	174.4	Ribosomal iTag	1190879	1190881	Phytoplankton community itags pl1	178256		511313	PRJNA511313	SRP174769	SAMN10636945	Gp0358285	Illumina MiSeq	2 × 301	68,732,146	n/a
56_S	ocean_biome	ocean	water	Monterey Bay, CA, USA	11/9/16	36.835 N, 121.901 W	174.4	Ribosomal iTag	1190880	1190883	Bacterial community itags	178344		511190	PRJNA511190	SRP174707	SAMN10636909	Gp0358379	Illumina MiSeq	2 × 301	62,297,368	n/a
57_DF	ocean_biome	ocean	water	Monterey Bay, CA, USA	11/6/16	36.835 N, 121.901 W	550	Ribosomal iTag	1190879	1190881	Phytoplankton community itags pl1	178257		511314	PRJNA511314	SRP174835	SAMN10636607	Gp0358286	Illumina MiSeq	2 × 301	123,612,874	n/a
57_S	ocean_biome	ocean	water	Monterey Bay, CA, USA	11/6/16	36.835 N, 121.901 W	550	Ribosomal iTag	1190880	1190883	Bacterial community itags	178345		511191	PRJNA511191	SRP174704	SAMN10636907	Gp0358380	Illumina MiSeq	2 × 301	97,596,240	n/a
58_DF	ocean_biome	ocean	water	Monterey Bay, CA, USA	11/4/16	36.835 N, 121.901 W	400	Ribosomal iTag	1190879	1190881	Phytoplankton community itags pl1	178258		511315	PRJNA511315	SRP174834	SAMN10636943	Gp0358287	Illumina MiSeq	2 × 301	130,885,636	n/a
58_S	ocean_biome	ocean	water	Monterey Bay, CA, USA	11/4/16	36.835 N, 121.901 W	400	Ribosomal iTag	1190880	1190883	Bacterial community itags	178346		511192	PRJNA511192	SRP174705	SAMN10636602	Gp0358381	Illumina MiSeq	2 × 301	84,473,242	n/a
58r_DF	ocean_biome	ocean	water	Monterey Bay, CA, USA	11/4/16	36.835 N, 121.901 W	425	Ribosomal iTag	1190879	1190881	Phytoplankton community itags pl1	178289		511214	PRJNA511214	SRP174783	SAMN10636637	Gp0358319	Illumina MiSeq	2 × 301	118,631,926	n/a
58r_S	ocean_biome	ocean	water	Monterey Bay, CA, USA	11/4/16	36.835 N, 121.901 W	425	Ribosomal iTag	1190880	1190883	Bacterial community itags	178377		511161	PRJNA511161	SRP174758	SAMN10636613	Gp0358414	Illumina MiSeq	2 × 301	371,434	n/a
59_DF	ocean_biome	ocean	water	Monterey Bay, CA, USA	10/21/16	36.835 N, 121.901 W	200	Ribosomal iTag	1190879	1190881	Phytoplankton community itags pl1	178259		511316	PRJNA511316	SRP174829	SAMN10636649	Gp0358288	Illumina MiSeq	2 × 301	70,527,912	n/a
59_S	ocean_biome	ocean	water	Monterey Bay, CA, USA	10/21/16	36.835 N, 121.901 W	200	Ribosomal iTag	1190880	1190883	Bacterial community itags	178347		511193	PRJNA511193	SRP174703	SAMN10636681	Gp0358382	Illumina MiSeq	2 × 301	80,643,318	n/a
60_DF	ocean_biome	ocean	water	Monterey Bay, CA, USA	11/12/16	36.835 N, 121.901 W	475	Ribosomal iTag	1190879	1190881	Phytoplankton community itags pl1	178260		511317	PRJNA511317	SRP174761	SAMN10636648	Gp0358289	Illumina MiSeq	2 × 301	94,567,578	n/a
60_S	ocean_biome	ocean	water	Monterey Bay, CA, USA	11/12/16	36.835 N, 121.901 W	475	Ribosomal iTag	1190880	1190883	Bacterial community itags	178348		511194	PRJNA511194	SRP174695	SAMN10636622	Gp0358383	Illumina MiSeq	2 × 301	69,084,316	n/a
61_DF	ocean_biome	ocean	water	Monterey Bay, CA, USA	10/31/16	36.835 N, 121.901 W	290	Ribosomal iTag	1190879	1190881	Phytoplankton community itags pl1	178261		511318	PRJNA511318	SRP174765	SAMN10636941	Gp0358290	Illumina MiSeq	2 × 301	68,405,862	n/a
61_S	ocean_biome	ocean	water	Monterey Bay, CA, USA	10/31/16	36.835 N, 121.901 W	290	Ribosomal iTag	1190880	1190883	Bacterial community itags	178349		511195	PRJNA511195	SRP174689	SAMN10636905	Gp0358384	Illumina MiSeq	2 × 301	84,761,600	n/a
62_DF	ocean_biome	ocean	water	Monterey Bay, CA, USA	10/28/16	36.835 N, 121.901 W	250	Ribosomal iTag	1190879	1190881	Phytoplankton community itags pl1	178262		511319	PRJNA511319	SRP174764	SAMN10636608	Gp0358291	Illumina MiSeq	2 × 301	53,843,482	n/a
62_S	ocean_biome	ocean	water	Monterey Bay, CA, USA	10/28/16	36.835 N, 121.901 W	250	Ribosomal iTag	1190880	1190883	Bacterial community itags	178350		511196	PRJNA511196	SRP174688	SAMN10636621	Gp0358385	Illumina MiSeq	2 × 301	71,817,998	n/a
63_DF	ocean_biome	ocean	water	Monterey Bay, CA, USA	10/24/16	36.835 N, 121.901 W	250	Ribosomal iTag	1190879	1190881	Phytoplankton community itags pl1	178263		511320	PRJNA511320	SRP174766	SAMN10636684	Gp0358292	Illumina MiSeq	2 × 301	246,218	n/a
63_S	ocean_biome	ocean	water	Monterey Bay, CA, USA	10/24/16	36.835 N, 121.901 W	250	Ribosomal iTag	1190880	1190883	Bacterial community itags	178351		511197	PRJNA511197	SRP174749	SAMN10636903	Gp0358387	Illumina MiSeq	2 × 301	54,614,042	n/a
64_DF	ocean_biome	ocean	water	Monterey Bay, CA, USA	10/5/16	36.835 N, 121.901 W	199.2	Ribosomal iTag	1190879	1190881	Phytoplankton community itags pl1	178264		511321	PRJNA511321	SRP174767	SAMN10636647	Gp0358293	Illumina MiSeq	2 × 301	88,243,568	n/a
64_S	ocean_biome	ocean	water	Monterey Bay, CA, USA	10/5/16	36.835 N, 121.901 W	199.2	Ribosomal iTag	1190880	1190883	Bacterial community itags	178352		511198	PRJNA511198	SRP174750	SAMN10637058	Gp0358388	Illumina MiSeq	2 × 301	57,253,812	n/a
65_DF	ocean_biome	ocean	water	Monterey Bay, CA, USA	11/2/16	36.835 N, 121.901 W	400	Ribosomal iTag	1190879	1190881	Phytoplankton community itags pl1	178265		511322	PRJNA511322	SRP174763	SAMN10636686	Gp0358294	Illumina MiSeq	2 × 301	122,270,414	n/a
65_S	ocean_biome	ocean	water	Monterey Bay, CA, USA	11/2/16	36.835 N, 121.901 W	400	Ribosomal iTag	1190880	1190883	Bacterial community itags	178353		511199	PRJNA511199	SRP174751	SAMN10636682	Gp0358389	Illumina MiSeq	2 × 301	91,420,924	n/a
66_DF	ocean_biome	ocean	water	Monterey Bay, CA, USA	10/3/16	36.835 N, 121.901 W	250	Ribosomal iTag	1190879	1190881	Phytoplankton community itags pl1	178266		511323	PRJNA511323	SRP174762	SAMN10636646	Gp0358295	Illumina MiSeq	2 × 301	123,413,010	n/a
66_S	ocean_biome	ocean	water	Monterey Bay, CA, USA	10/3/16	36.835 N, 121.901 W	250	Ribosomal iTag	1190880	1190883	Bacterial community itags	178354		511200	PRJNA511200	SRP174752	SAMN10636901	Gp0358390	Illumina MiSeq	2 × 301	53,405,226	n/a
67_DF	ocean_biome	ocean	water	Monterey Bay, CA, USA	11/15/16	36.835 N, 121.901 W	1000	Ribosomal iTag	1190879	1190881	Phytoplankton community itags pl1	178267		511324	PRJNA511324	SRP174772	SAMN10636713	Gp0358296	Illumina MiSeq	2 × 301	101,911,978	n/a
67_S	ocean_biome	ocean	water	Monterey Bay, CA, USA	11/15/16	36.835 N, 121.901 W	1000	Ribosomal iTag	1190880	1190883	Bacterial community itags	178355		511201	PRJNA511201	SRP174698	SAMN10637059	Gp0358391	Illumina MiSeq	2 × 301	85,830,150	n/a
68_DF	ocean_biome	ocean	water	Monterey Bay, CA, USA	11/7/16	36.835 N, 121.901 W	575	Ribosomal iTag	1190879	1190881	Phytoplankton community itags pl1	178268		511325	PRJNA511325	SRP174771	SAMN10636703	Gp0358297	Illumina MiSeq	2 × 301	118,754,734	n/a
68_S	ocean_biome	ocean	water	Monterey Bay, CA, USA	11/7/16	36.835 N, 121.901 W	575	Ribosomal iTag	1190880	1190883	Bacterial community itags	178356		511202	PRJNA511202	SRP174692	SAMN10636620	Gp0358392	Illumina MiSeq	2 × 301	69,421,436	n/a
7_DF	ocean_biome	ocean	water	Monterey Bay, CA, USA	10/26/16	36.835 N, 121.901 W	200	Ribosomal iTag	1190879	1190881	Phytoplankton community itags pl1	178208		511265	PRJNA511265	SRP174805	SAMN10636663	Gp0358234	Illumina MiSeq	2 × 301	101,529,708	n/a
7_S	ocean_biome	ocean	water	Monterey Bay, CA, USA	10/26/16	36.835 N, 121.901 W	200	Ribosomal iTag	1190880	1190883	Bacterial community itags	178296		511221	PRJNA511221	SRP174682	SAMN10636924	Gp0358327	Illumina MiSeq	2 × 301	70,919,814	n/a
70_DF	ocean_biome	ocean	water	Monterey Bay, CA, USA	10/7/16	36.835 N, 121.901 W	207.5	Ribosomal iTag	1190879	1190881	Phytoplankton community itags pl1	178269		511326	PRJNA511326	SRP174777	SAMN10636645	Gp0358298	Illumina MiSeq	2 × 301	118,522,964	n/a
70_S	ocean_biome	ocean	water	Monterey Bay, CA, USA	10/7/16	36.835 N, 121.901 W	207.5	Ribosomal iTag	1190880	1190883	Bacterial community itags	178357		511203	PRJNA511203	SRP174690	SAMN10636683	Gp0358393	Illumina MiSeq	2 × 301	77,110,180	n/a
71_DF	ocean_biome	ocean	water	Monterey Bay, CA, USA	10/18/16	36.835 N, 121.901 W	200	Ribosomal iTag	1190879	1190881	Phytoplankton community itags pl1	178270		511327	PRJNA511327	SRP174770	SAMN10636687	Gp0358299	Illumina MiSeq	2 × 301	75,922,434	n/a
71_S	ocean_biome	ocean	water	Monterey Bay, CA, USA	10/18/16	36.835 N, 121.901 W	200	Ribosomal iTag	1190880	1190883	Bacterial community itags	178358		511204	PRJNA511204	SRP174693	SAMN10636899	Gp0358394	Illumina MiSeq	2 × 301	72,771,566	n/a
73_DF	ocean_biome	ocean	water	Monterey Bay, CA, USA	11/16/16	36.835 N, 121.901 W	1000	Ribosomal iTag	1190879	1190881	Phytoplankton community itags pl1	178271		511328	PRJNA511328	SRP174776	SAMN10636644	Gp0358300	Illumina MiSeq	2 × 301	43,210,958	n/a
73_S	ocean_biome	ocean	water	Monterey Bay, CA, USA	11/16/16	36.835 N, 121.901 W	1000	Ribosomal iTag	1190880	1190883	Bacterial community itags	178359		511205	PRJNA511205	SRP174691	SAMN10636897	Gp0358395	Illumina MiSeq	2 × 301	58,997,806	n/a
74_DF	ocean_biome	ocean	water	Monterey Bay, CA, USA	11/11/16	36.835 N, 121.901 W	425	Ribosomal iTag	1190879	1190881	Phytoplankton community itags pl1	178272		511329	PRJNA511329	SRP174774	SAMN10636688	Gp0358301	Illumina MiSeq	2 × 301	96,386,822	n/a
74_S	ocean_biome	ocean	water	Monterey Bay, CA, USA	11/11/16	36.835 N, 121.901 W	425	Ribosomal iTag	1190880	1190883	Bacterial community itags	178360		511206	PRJNA511206	SRP174696	SAMN10636895	Gp0358396	Illumina MiSeq	2 × 301	61,698,378	n/a
75_DF	ocean_biome	ocean	water	Monterey Bay, CA, USA	11/14/16	36.835 N, 121.901 W	825	Ribosomal iTag	1190879	1190881	Phytoplankton community itags pl1	178273		511330	PRJNA511330	SRP174775	SAMN10636643	Gp0358302	Illumina MiSeq	2 × 301	112,452,998	n/a
75_S	ocean_biome	ocean	water	Monterey Bay, CA, USA	11/14/16	36.835 N, 121.901 W	825	Ribosomal iTag	1190880	1190883	Bacterial community itags	178361		511242	PRJNA511242	SRP174694	SAMN10636671	Gp0358397	Illumina MiSeq	2 × 301	94,110,660	n/a
76_DF	ocean_biome	ocean	water	Monterey Bay, CA, USA	11/10/16	36.835 N, 121.901 W	350	Ribosomal iTag	1190879	1190881	Phytoplankton community itags pl1	178274		511331	PRJNA511331	SRP174760	SAMN10636939	Gp0358304	Illumina MiSeq	2 × 301	105,368,662	n/a
76_S	ocean_biome	ocean	water	Monterey Bay, CA, USA	11/10/16	36.835 N, 121.901 W	350	Ribosomal iTag	1190880	1190883	Bacterial community itags	178362		511243	PRJNA511243	SRP174697	SAMN10636893	Gp0358398	Illumina MiSeq	2 × 301	81,985,176	n/a
77_DF	ocean_biome	ocean	water	Monterey Bay, CA, USA	10/7/16	36.835 N, 121.901 W	201.5	Ribosomal iTag	1190879	1190881	Phytoplankton community itags pl1	178275		511253	PRJNA511253	SRP174842	SAMN10636599	Gp0358305	Illumina MiSeq	2 × 301	59,957,394	n/a
77_S	ocean_biome	ocean	water	Monterey Bay, CA, USA	10/7/16	36.835 N, 121.901 W	201.5	Ribosomal iTag	1190880	1190883	Bacterial community itags	178363		511244	PRJNA511244	SRP174743	SAMN10636611	Gp0358399	Illumina MiSeq	2 × 301	95,181,618	n/a
78_DF	ocean_biome	ocean	water	Monterey Bay, CA, USA	9/26/16	36.835 N, 121.901 W	92.5	Ribosomal iTag	1190879	1190881	Phytoplankton community itags pl1	178276		511254	PRJNA511254	SRP174802	SAMN10636642	Gp0358306	Illumina MiSeq	2 × 301	54,360,600	n/a
78_S	ocean_biome	ocean	water	Monterey Bay, CA, USA	9/26/16	36.835 N, 121.901 W	92.5	Ribosomal iTag	1190880	1190883	Bacterial community itags	178364		511245	PRJNA511245	SRP174744	SAMN10636619	Gp0358400	Illumina MiSeq	2 × 301	61,418,448	n/a
79_DF	ocean_biome	ocean	water	Monterey Bay, CA, USA	11/7/16	36.835 N, 121.901 W	550	Ribosomal iTag	1190879	1190881	Phytoplankton community itags pl1	178277		511255	PRJNA511255	SRP174788	SAMN10636937	Gp0358307	Illumina MiSeq	2 × 301	131,771,780	n/a
79_S	ocean_biome	ocean	water	Monterey Bay, CA, USA	11/7/16	36.835 N, 121.901 W	550	Ribosomal iTag	1190880	1190883	Bacterial community itags	178365		511246	PRJNA511246	SRP174746	SAMN10636891	Gp0358401	Illumina MiSeq	2 × 301	100,075,276	n/a
8_DF	ocean_biome	ocean	water	Monterey Bay, CA, USA	11/5/16	36.835 N, 121.901 W	775	Ribosomal iTag	1190879	1190881	Phytoplankton community itags pl1	178209		511266	PRJNA511266	SRP174804	SAMN10636662	Gp0358235	Illumina MiSeq	2 × 301	123,461,772	n/a
8_S	ocean_biome	ocean	water	Monterey Bay, CA, USA	11/5/16	36.835 N, 121.901 W	775	Ribosomal iTag	1190880	1190883	Bacterial community itags	178297		511222	PRJNA511222	SRP174678	SAMN10637053	Gp0358328	Illumina MiSeq	2 × 301	80,934,084	n/a
80_DF	ocean_biome	ocean	water	Monterey Bay, CA, USA	10/11/16	36.835 N, 121.901 W	100	Ribosomal iTag	1190879	1190881	Phytoplankton community itags pl1	178278		511256	PRJNA511256	SRP174789	SAMN10636935	Gp0358308	Illumina MiSeq	2 × 301	95,350,780	n/a
80_S	ocean_biome	ocean	water	Monterey Bay, CA, USA	10/11/16	36.835 N, 121.901 W	100	Ribosomal iTag	1190880	1190883	Bacterial community itags	178366		511247	PRJNA511247	SRP174745	SAMN10636890	Gp0358402	Illumina MiSeq	2 × 301	57,104,516	n/a
81_DF	ocean_biome	ocean	water	Monterey Bay, CA, USA	10/11/16	36.835 N, 121.901 W	100	Ribosomal iTag	1190879	1190881	Phytoplankton community itags pl1	178279		511257	PRJNA511257	SRP174786	SAMN10636600	Gp0358309	Illumina MiSeq	2 × 301	79,994,362	n/a
81_S	ocean_biome	ocean	water	Monterey Bay, CA, USA	10/11/16	36.835 N, 121.901 W	100	Ribosomal iTag	1190880	1190883	Bacterial community itags	178367		511248	PRJNA511248	SRP174739	SAMN10636612	Gp0358403	Illumina MiSeq	2 × 301	62,038,508	n/a
82_DF	ocean_biome	ocean	water	Monterey Bay, CA, USA	10/10/16	36.835 N, 121.901 W	100	Ribosomal iTag	1190879	1190881	Phytoplankton community itags pl1	178280		511258	PRJNA511258	SRP174806	SAMN10636933	Gp0358310	Illumina MiSeq	2 × 301	107,646,028	n/a
82_S	ocean_biome	ocean	water	Monterey Bay, CA, USA	10/10/16	36.835 N, 121.901 W	100	Ribosomal iTag	1190880	1190883	Bacterial community itags	178368		511249	PRJNA511249	SRP174741	SAMN10636888	Gp0358404	Illumina MiSeq	2 × 301	34,461,490	n/a
84_DF	ocean_biome	ocean	water	Monterey Bay, CA, USA	10/11/16	36.835 N, 121.901 W	100	Ribosomal iTag	1190879	1190881	Phytoplankton community itags pl1	178281		511259	PRJNA511259	SRP174840	SAMN10636641	Gp0358311	Illumina MiSeq	2 × 301	87,418,226	n/a
84_S	ocean_biome	ocean	water	Monterey Bay, CA, USA	10/11/16	36.835 N, 121.901 W	100	Ribosomal iTag	1190880	1190883	Bacterial community itags	178369		511250	PRJNA511250	SRP174742	SAMN10636690	Gp0358405	Illumina MiSeq	2 × 301	92,202,922	n/a
85_DF	ocean_biome	ocean	water	Monterey Bay, CA, USA	10/13/16	36.835 N, 121.901 W	100	Ribosomal iTag	1190879	1190881	Phytoplankton community itags pl1	178282		511207	PRJNA511207	SRP174812	SAMN10637049	Gp0358312	Illumina MiSeq	2 × 301	394,310	n/a
85_S	ocean_biome	ocean	water	Monterey Bay, CA, USA	10/13/16	36.835 N, 121.901 W	100	Ribosomal iTag	1190880	1190883	Bacterial community itags	178370		511251	PRJNA511251	SRP174757	SAMN10636618	Gp0358406	Illumina MiSeq	2 × 301	84,782,670	n/a
89_DF	ocean_biome	ocean	water	Monterey Bay, CA, USA	11/5/16	36.835 N, 121.901 W	575	Ribosomal iTag	1190879	1190881	Phytoplankton community itags pl1	178283		511208	PRJNA511208	SRP174778	SAMN10636932	Gp0358313	Illumina MiSeq	2 × 301	17,458	n/a
89_S	ocean_biome	ocean	water	Monterey Bay, CA, USA	11/5/16	36.835 N, 121.901 W	575	Ribosomal iTag	1190880	1190883	Bacterial community itags	178371		511252	PRJNA511252	SRP174755	SAMN10636617	Gp0358407	Illumina MiSeq	2 × 301	62,152,286	n/a
9_DF	ocean_biome	ocean	water	Monterey Bay, CA, USA	11/15/16	36.835 N, 121.901 W	975	Ribosomal iTag	1190879	1190881	Phytoplankton community itags pl1	178210		511267	PRJNA511267	SRP174807	SAMN10636661	Gp0358236	Illumina MiSeq	2 × 301	122,328,808	n/a
9_S	ocean_biome	ocean	water	Monterey Bay, CA, USA	11/15/16	36.835 N, 121.901 W	975	Ribosomal iTag	1190880	1190883	Bacterial community itags	178298		511223	PRJNA511223	SRP174683	SAMN10636636	Gp0358329	Illumina MiSeq	2 × 301	81,732,336	n/a
90_DF	ocean_biome	ocean	water	Monterey Bay, CA, USA	11/8/16	36.835 N, 121.901 W	175	Ribosomal iTag	1190879	1190881	Phytoplankton community itags pl1	178284		511209	PRJNA511209	SRP174779	SAMN10636640	Gp0358314	Illumina MiSeq	2 × 301	94,883,026	n/a
90_S	ocean_biome	ocean	water	Monterey Bay, CA, USA	11/8/16	36.835 N, 121.901 W	175	Ribosomal iTag	1190880	1190883	Bacterial community itags	178372		511156	PRJNA511156	SRP174756	SAMN10637063	Gp0358408	Illumina MiSeq	2 × 301	37,095,842	n/a
91_DF	ocean_biome	ocean	water	Monterey Bay, CA, USA	11/11/16	36.835 N, 121.901 W	475	Ribosomal iTag	1190879	1190881	Phytoplankton community itags pl1	178285		511210	PRJNA511210	SRP174780	SAMN10636672	Gp0358315	Illumina MiSeq	2 × 301	104,717,900	n/a
91_S	ocean_biome	ocean	water	Monterey Bay, CA, USA	11/11/16	36.835 N, 121.901 W	475	Ribosomal iTag	1190880	1190883	Bacterial community itags	178373		511157	PRJNA511157	SRP174754	SAMN10636616	Gp0358409	Illumina MiSeq	2 × 301	76,699,014	n/a
